# Retinoic acid and TGF-β signalling cooperate to overcome MYCN-induced retinoid resistance

**DOI:** 10.1186/s13073-017-0407-3

**Published:** 2017-02-10

**Authors:** David J. Duffy, Aleksandar Krstic, Melinda Halasz, Thomas Schwarzl, Anja Konietzny, Kristiina Iljin, Desmond G. Higgins, Walter Kolch

**Affiliations:** 1 Systems Biology Ireland, University College Dublin, Belfield, Dublin 4 Ireland; 2 Conway Institute of Biomolecular & Biomedical Research, University College Dublin, Belfield, Dublin 4 Ireland; 3 School of Medicine, University College Dublin, Belfield, Dublin 4 Ireland; 4 The Whitney Laboratory for Marine Bioscience and Sea Turtle Hospital, University of Florida, St. Augustine, Florida 32080 USA; 5 European Molecular Biology Laboratory (EMBL), Meyerhofstraße 1, 69117 Heidelberg, Germany; 6 Present address: Department of Biology, University of Konstanz, Konstanz, Germany; 7 VTT Technical Research Centre of Finland, Tietotie 2, FI-02044 VTT, Espoo, Finland

**Keywords:** Neuroblastoma, Kartogenin (KGN), RepSox, MYC (c-MYC), Differentiation, mRNA sequencing (mRNA-seq), ChIP sequencing (ChIP-seq), Transforming growth factor beta (TGF-β) signalling, Interaction proteomics, Precision medicine, Genome medicine, Wnt β-catenin signalling, Cancer, Systems medicine, Neuronal differentiation, Differentiation therapy

## Abstract

**Background:**

Retinoid therapy is widely employed in clinical oncology to differentiate malignant cells into their more benign counterparts. However, certain high-risk cohorts, such as patients with MYCN-amplified neuroblastoma, are innately resistant to retinoid therapy. Therefore, we employed a precision medicine approach to globally profile the retinoid signalling response and to determine how an excess of cellular MYCN antagonises these signalling events to prevent differentiation and confer resistance.

**Methods:**

We applied RNA sequencing (RNA-seq) and interaction proteomics coupled with network-based systems level analysis to identify targetable vulnerabilities of MYCN-mediated retinoid resistance. We altered MYCN expression levels in a MYCN-inducible neuroblastoma cell line to facilitate or block retinoic acid (RA)-mediated neuronal differentiation. The relevance of differentially expressed genes and transcriptional regulators for neuroblastoma outcome were then confirmed using existing patient microarray datasets.

**Results:**

We determined the signalling networks through which RA mediates neuroblastoma differentiation and the inhibitory perturbations to these networks upon MYCN overexpression. We revealed opposing regulation of RA and MYCN on a number of differentiation-relevant genes, including LMO4, CYP26A1, ASCL1, RET, FZD7 and DKK1. Furthermore, we revealed a broad network of transcriptional regulators involved in regulating retinoid responsiveness, such as Neurotrophin, PI3K, Wnt and MAPK, and epigenetic signalling. Of these regulators, we functionally confirmed that MYCN-driven inhibition of transforming growth factor beta (TGF-β) signalling is a vulnerable node of the MYCN network and that multiple levels of cross-talk exist between MYCN and TGF-β. Co-targeting of the retinoic acid and TGF-β pathways, through RA and kartogenin (KGN; a TGF-β signalling activating small molecule) combination treatment, induced the loss of viability of MYCN-amplified retinoid-resistant neuroblastoma cells.

**Conclusions:**

Our approach provides a powerful precision oncology tool for identifying the driving signalling networks for malignancies not primarily driven by somatic mutations, such as paediatric cancers. By applying global omics approaches to the signalling networks regulating neuroblastoma differentiation and stemness, we have determined the pathways involved in the MYCN-mediated retinoid resistance, with TGF-β signalling being a key regulator. These findings revealed a number of combination treatments likely to improve clinical response to retinoid therapy, including co-treatment with retinoids and KGN, which may prove valuable in the treatment of high-risk MYCN-amplified neuroblastoma.

**Electronic supplementary material:**

The online version of this article (doi:10.1186/s13073-017-0407-3) contains supplementary material, which is available to authorized users.

## Background

The paediatric cancer neuroblastoma arises when neuronal stem cells of a transient embryonal tissue, the neural crest, fail to complete their terminal differentiation into neurons of the peripheral nervous system and instead transform to become oncogenic [1, 2]. This failure of an embryonal population of neuroblasts to differentiate is largely due to the aberrant maintenance of stemness signals arising from genetic and epigenetic lesions [3–7]. Therefore, neuroblastoma has been termed a malignancy which is due to a differentiation block [8, 9], with tumours consisting of more stem-like cells representing more aggressive and high-risk disease [10, 11]. Interestingly, neuroblastoma has the highest rate of spontaneous regression of any solid tumour [12–15], which is thought to be due to the late restoration of normal developmental signalling resulting in tumour cell differentiation or apoptosis. Thus, even neuroblastomas with metastatic spread (stage 4S) can have a good prognosis due to spontaneous regression, especially in infants below 18 months of age [16, 17]. By contrast, neuroblastomas diagnosed later than 18 months or those with amplification of the MYCN oncogene have much worse prognosis. In fact, neuroblastoma accounts for 15% of all childhood cancer deaths, the highest of any solid tumour [16]. The survival rate of the high-risk neuroblastoma patients has not improved in decades, remaining steady at a 5-year survival of 40–50% [18]. Therefore, it is a clinical imperative that effective therapeutics for high-risk neuroblastoma be identified.

Differentiation therapy is widely employed in clinical cancer treatment [19–26] as both primary and maintenance therapies. Retinoid-induced differentiation of neuronal precursors is a successful treatment strategy for low and intermediate risk neuroblastoma patients. Both all-trans-retinoic acid (ATRA, tretinoin) and 13-cis-retinoic acid (isotretinoin) are used in neuroblastoma therapy with isotretinoin being the preferred compound [20–22, 24, 27, 28]. However, despite the effectiveness of retinoid treatment for some patients, it is ineffective for many high-risk patients [24, 29]. This is largely due to MYCN-induced resistance to retinoid therapy [24]. MYCN is a primarily neuronal-specific member of the MYC proto-oncogene family. It is expressed during normal neural crest development, and when under normal regulation it does not prevent terminal differentiation of neuroblasts [30, 31]. However, amplified MYCN prevents neuronal differentiation [32, 33], and aberrant MYCN signalling alone is sufficient to induce neuroblastoma in animal models [2, 34]. MYCN amplification occurs in over 20% of neuroblastoma and contributes to metastasis and chemoresistance [17, 35]. Additionally, 50% of neuroblastoma patients who initially respond to retinoic acid (RA) therapy develop retinoid resistance [8]. However, despite the variability of the clinical response to RA, there is a lack of recurrent somatic mutations in components of the RA signalling pathway [3, 36], suggesting adaptor mechanisms conveying resistance. Furthermore, epigenetic regulation has been shown to block neuronal precursor differentiation [37]. Therefore, rather than focussing on mutations or epigenetic status, we employed RNA-seq and network-based analysis to globally profile the functional status of gene regulatory networks in response to RA. Analysis at this level captures the functional effects of all upstream regulatory alterations regardless of their origin (e.g. mutations, transcriptional and epigenetic alterations, miRNA/lncRNA-mediated regulation). Such approaches are particularly pertinent for paediatric cancers as they have a low rate of genetic mutation compared with adult cancers, and accordingly present far fewer actionable genetic alterations [38]. The only actionable mutation in neuroblastoma is mutant ALK, but even this gene is mutated in only 9.2% of tumours [3, 38].

While elevated MYCN levels inhibit RA-mediated neuronal differentiation [32, 33], the precise molecular mechanisms are unknown. Elucidating these mechanisms could reveal potential targets and combination treatments that can re-sensitise MYCN-amplified cells to retinoid therapy. To this end, we transcriptionally profiled (RNA-seq) ATRA (from here on referred to as RA) exposed neuroblastoma cells in the presence of high and low MYCN expression levels and performed network-based precision medicine analysis [38–41]. Additionally, we profiled changes in the MYCN protein–protein interactome in response to RA. We thereby identified how MYCN globally modulates the differentiation response and identified network nodes which can be therapeutically targeted to prevent amplified MYCN from inhibiting the RA-induced differentiation of neuroblastoma cells.

## Methods

### Cell culture and inhibitor treatments

The cell line SH-SY5Y/6TR(EU)/pTrex-Dest-30/MYCN (SY5Y-MYCN) stably expresses a tetracycline-inducible MYCN expression vector and was kindly provided by the Westermann lab [42–44]. Cells were cultured in RPMI 1640 (Gibco) supplemented with 10% fetal bovine serum (Gibco), 2 mM L-glutamine (Gibco) and 1% penicillin-streptomycin solution (Gibco). RA (Sigma-Aldrich) was given at a final concentration of 1 μM, to induce differentiation. Kartogenin (Selleck) was dissolved in DMSO and used in concentrations ranging from 0.1–20 μM. RepSox (Selleck) was dissolved in DMSO and used in concentrations ranging from 1–100 nM. For vehicle controls equivalent volumes of DMSO were added to cells. Doxycycline (Dox; Sigma) dissolved in water was used at a final concentration of 1 μg/ml to induce MYCN expression in SY5Y-MYCN. Dox was replenished every 24 h for any treatment longer than a 24 h duration. For Dox co-treatments cells were pre-incubated with doxycycline for 24 h before addition of RA and had fresh doxycycline given at the start of RA treatment. Cells were imaged using an Olympus CKX41 microscope.

### Western blot and quantitative RT-PCR

Western blotting and quantitative RT-PCR (RT-qPCR) were performed as previously described [45]. Antibodies used were TrkB (1/1,000 dilution, #4603, Cell Signaling Technologies) and ERK1/2 (1/10,000 dilution, M5670, Sigma Aldrich). TaqMan assays (Applied Biosystems) used were DKK1 (Hs00183740_m1*), EGR1 (Hs00152928_m1*), FZD7 (Hs00275833_s1*), RET (Hs01120030_m1*), NTRK2 (Hs00178811_m1*), c-MYC (Hs00905030_m1*), MYCN (Hs00232074_m1*), ASCL1 (Hs04187546_g1), LMO4 (Hs01086790_m1) and the endogenous control genes RPLPO (4310879E) and ACTB (β-actin, 4326315E). Biological duplicates were generated for all samples; technical replicates for every sample were also performed.

### mRNA sequencing and bioinformatics analysis

SY5Y-MYCN cells were exposed to one of four treatments (24 h DMSO, 24 h RA, 24 h RA and 48 h Dox and 48 h Dox), with biological duplicates. While the 48-h Dox-only sample was also previously analysed as part of a MYCN overexpression time-course [42], analysis of the RA samples and their comparison with the 48-h Dox only samples has not previously been published. The 48-h Dox treatment was performed and the samples were sequenced together with the DMSO, RA-only and RA and Dox samples. Total mRNA was extracted using TRI Reagent (Sigma-Aldrich) according to the manufacturer’s protocol, and DNA was digested with RQ1 RNase free DNase (Promega). RNA quality was checked by RT-qPCR (as above) and on a 2100 Bioanalyzer (Agilent) using a Eukaryote Total RNA Nano Chip (version 2.6). All samples had a RIN value in the range of 8.40–9.20. Sequencing libraries were generated from 2 μg of total RNA per sample with a TruSeq RNA sample preparation Kit v2 (Illumina) according to the manufacturer’s protocol. Size and purity of the libraries were analysed on a Bioanalyzer High Sensitivity DNA chip (Agilent). Libraries were clustered using TruSeq Single-Read Cluster Kit v5-CS-GA (Illumina) and sequenced on an Illumina Genome Analyzer IIx with a TruSeq SBS Kit v5-GA (Illumina).

The sequence reads were analysed as described previously [45]. Differentially expressed genes were called using general linear models in edgeR [46]. *P* values were adjusted for multiple testing with the Benjamini–Hochberg correction and a corrected P cutoff of 0.05 was used. To make the absolute expression levels of genes comparable with each other, the read counts per million were adjusted by gene length in kilobases (CPMkb). The mRNA-seq data were deposited in ArrayExpress (http://www.ebi.ac.uk/arrayexpress) under accession number E-MTAB-2689.

#### Additional software tools

Ingenuity Pathway Analysis (IPA) software was also used for the inferred transcriptional regulator (ITR), pathway and gene ontology (GO) analysis. String (http://www.string-db.org/) was used to generate protein–protein interaction networks, and the KEGG pathway enrichment analysis tool in String was also applied to these networks. Area-proportional Venn diagrams were generated using BioVenn (http://www.cmbi.ru.nl/cdd/biovenn/) and four-way comparisons were generated using Venny (http://bioinfogp.cnb.csic.es/tools/venny/index.html). Measurements of neurite length and cell width were obtained from images using ImageJ v1.44p (http://imagej.nih.gov/ij).

### Proteomics

Mass spectrometry-based interaction proteomics were conducted on SY5Y-MYCN (un-induced, 48-h MYCN overexpression, 24-h 1-μM RA treatment and 48-h MYCN overexpression and 24-h 1-μM RA co-treatment) for the MYCN protein. Interaction proteomics were performed as previously described [47]. MYCN was immunoprecipitated by using Protein A/G PLUS-agarose beads (sc-2003, Santa Cruz) conjugated to MYCN antibody (1/1,000 dilution, sc-53993, Santa Cruz) or IgG. Three biological and two technical replicates were performed per condition.

### Cell viability assay

Cell viability was analysed by MTS assay as described [45], with values normalised to untreated control cells. The results represent the mean ± standard deviation of triplicate biological replicates, expressed as a percentage of control.

## Results

### MYCN overexpression inhibits RA-induced neuronal differentiation

**Fig. 1:**
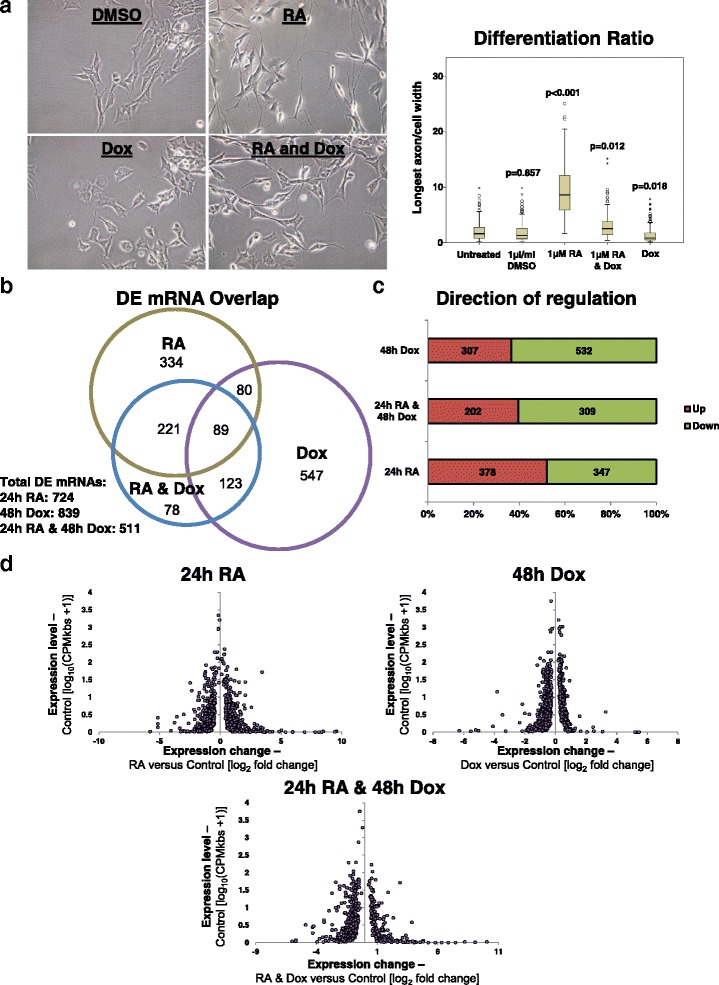
MYCN overexpression antagonises RA-induced differentiation of neuroblastoma cells at the transcriptional level. **a** SY5Y-MYCN cells treated for the following time-periods: 1 μl/ml DMSO for 3 days, 1 μg/ml Dox (to induce MYCN expression) for 4 days, 1 μM RA for 3 days or combination treatment 1 μg/ml Dox for 4 days and 1 μg/ml RA for 3 days. *Left*: Imaging of the SY5Y-MYCN cells. All panels are imaged at 40× magnification. *Right*: The differentiation ratio of cells treated with individual agents or combination treatments was calculated by dividing the length of the longest axon of a cell by the cell’s width. The images from three biological replicates were pooled, and then measurements of individual cells were made using ImageJ v1.44p (http://imagej.nih.gov/ij). The range of measured cells (N) per treatment group is 150–259. The differentiation ratio of each cell was then calculated by dividing the length of its longest axon by the cell’s width. The *p* value for each treatment group compared with untreated control cells (*t*-test) is shown above each treatment box. **b** Number and overlap of differentially expressed (*DE*) genes in each treatment group, as detected by RNA-seq. The area-proportional Venn diagram was generated using BioVenn (http://www.cmbi.ru.nl/cdd/biovenn/) [123] and shows the overlap between the genes DE in each treatment group compared to the untreated control cells. **c** Proportions of up- and downregulated DE mRNAs for each treatment group when compared with untreated SY5Y-MYCN cells, as detected by RNA-seq. **d** All significant DE genes (RNA-seq), with expression level in the control state (no RA and un-induced MYCN) plotted against the fold change after treatment. Each significant DE gene is denoted by a purple dot

SY5Y neuroblastoma cells treated with RA undergo neuronal differentiation to become dopaminergic neurons [45, 48–51]. We profiled global transcriptional changes mediated by RA in the MYCN Dox-inducible SY5Y-MYCN cell line, which was previously generated from the parental SY5Y cell line by the Westermann lab [42–44]. To assess the effect of MYCN overexpression on neuronal differentiation we imaged SY5Y-MYCN cells treated with RA while overexpression of the MYCN transgene was either induced or un-induced (Fig. 1a). A differentiation ratio for each treatment group was then calculated by dividing the length of the longest axon of a cell by the cell’s width. Like SY5Y cells, SY5Y-MYCN cells underwent RA-mediated differentiation in the absence of MYCN induction. However, when MYCN expression was induced (reaching 10–15 times higher levels than in un-induced cells; Additional file 1: Figure S1a) the ability of RA to efficiently differentiate these cells strongly and significantly was attenuated (*t*-test, RA versus RA and Dox *p* < 0.0001). While endogenous MYCN mRNA (parental SY5Y cells) expression was downregulated by RA treatment, ectopic MYCN in SY5Y-MYCN cell lines was not reduced as it is not under the control of the endogenous MYCN promoter (Additional file 1: Figure S1b; also see Duffy et al. [45]). Confirming that the RA was active, it reduced the expression of endogenous c-MYC mRNA by a similar extent in both SY5Y and un-induced SY5Y-MYCN cell lines (Additional file 1: Figure S1b).

### MYCN overexpression antagonises the normal transcriptional response to RA treatment

The mechanisms through which MYCN blocks RA-mediated neuronal differentiation are highly relevant to MYCN-amplified neuroblastoma patients, who generally do not respond well to retinoid treatment [20, 27]. Therefore, to identify these mechanisms we conducted mRNA-seq of SY5Y-MYCN cells under four treatment conditions: (i) 24-h DMSO (control), (ii) 24-h RA, (iii) 24-h RA and 48-h Dox, and (iv) 48-h Dox. Firstly, we confirmed that a number of the genes encoding the RA receptors, which are required to facilitate cellular responsiveness to RA, were expressed in SY5Y-MYCN cells (Additional file 1: Figure S1c). Of these the expression of both RARA and RARB was upregulated upon RA treatment. In total, between 511 and 839 differentially expressed (DE) genes were detected per treatment group, with a high degree of overlap between the co-treatment (RA and Dox) sample and the individual treatments (Fig. 1b; Additional file 2: Table S1; Additional file 3: Table S2; Additional file 4: Table S3; Additional file 5: Table S4). MYCN overexpression predominantly downregulated gene expression as we have previously described [42], while RA treatment produced a very similar number of up- and downregulated DE genes (Fig. 1c). While there was a trend for the greatest fold changes to occur in genes which had lower pre-treatment expression states, genes across the full range of pre-treatment expression levels were differentially expressed (Fig. 1d).

**Fig. 2:**
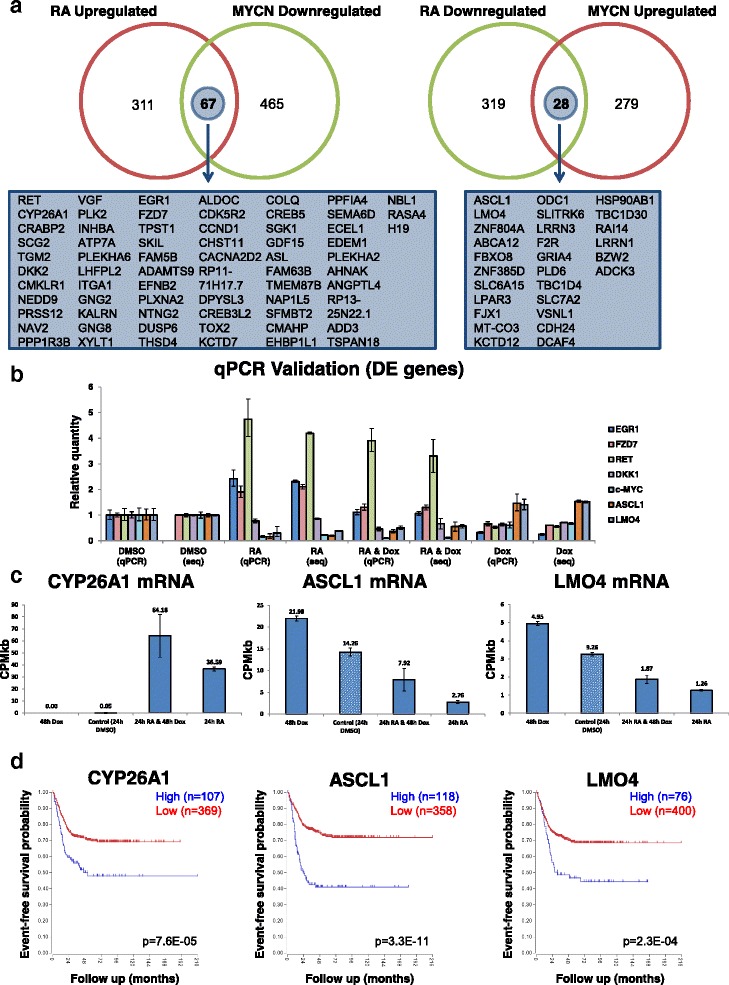
Likely drivers of the MYCN-mediated differentiation block: genes regulated in opposing directions by RA treatment and MYCN overexpression. **a** Number and overlap of the 169 differentially expressed genes (compared with control transcriptome) altered in opposing directions between the RA and MYCN overexpression treatments. *Left*: Overlap of genes upregulated by RA (24 h) and downregulated by MYCN overexpression (48 h). *Right*: Overlap of genes upregulated by MYCN overexpression (48 h) and downregulated by RA (24 h). The names of the genes which were common between the treatments but differentially expressed in opposing directions are shown below each Venn diagram. Venn diagrams were generated using Venny (http://bioinfogp.cnb.csic.es/tools/venny/index.html). **b** RT-qPCR validation in SY5Y-MYCN cells of the RNA-seq for a panel of differentially expressed genes, EGR1, FZD7, RET, DKK1,c-MYC, ASCL1 and LMO4. Levels of expression for each gene are set relative to those in untreated samples of control cells. RT-qPCR time-points mirror the RNA-seq time-points. RT-qPCR samples denoted by “*qPCR*“ with their RNA-seq counterparts denoted by “*seq*”. *Error bars* for qPCR samples denote RQ Min and RQ Max, while for RNA-seq samples they are standard deviation. **c** Levels of absolute gene expression for CYP26A1 (*left*), ASCL1 (*centre*) and LMO4 (*right*), in each of the SY5Y-MYCN RNA-seq treatments. Expression is in read counts per million adjusted by gene length in kilobases (*CPMkb*), with *error bars* denoting the standard deviation between replicates. **d** Kaplan–Meier survival curves showing the predictive strength of the expression levels of the CYP26A1 (*left*), ASCL1 (*centre*) and LMO4 (*right*) mRNAs in neuroblastoma tumours on patient outcome. Generated using the Kocak [69] 649 neuroblastoma tumour dataset in the R2: Genomics Analysis and Visualization Platform (http://r2.amc.nl)

Of the 169 genes regulated in common between MYCN overexpression and RA treatment, 95 were regulated in opposing directions (up- or downregulated) by each treatment (Fig. 2a). These differentially activated genes are likely key to MYCN’s ability to block RA-mediated neuronal differentiation and contain both known and novel components of differentiation signalling (see below). To validate the accuracy of the RNA-seq analysis we analysed the changes in expression (by qPCR) of MYCN (Additional file 1: Figure S1a) and seven selected genes, RET, DKK1, EGR1, FZD7, ASCL1, LMO4 and c-MYC (Fig. 2b), which were identified as being DE in the RNA-seq data. The results confirmed the reliability of the RNA sequencing data (Fig. 2b; Additional file 1: Figure S1a). The qPCR also confirmed the differing direction of regulation for RET, FZD7, EGR1, ASCL1 and LMO4 between the RA treatments and MYCN induction (Fig. 2b). To eliminate any Dox-related, non-MYCN-dependent, role in the expression changes of these genes we treated parental SY5Y cells with Dox. Dox treatment in SY5Y cells did not reproduce the expression changes observed when MYCN was overexpressed in SY5Y-MYCN cells via Dox treatment (Additional file 1: Figure S1d).

We also confirmed by qPCR and western blotting the strong induction of the BDNF receptor NTRK2 (TrkB) upon RA treatment, which was revealed in the RNA-seq results (Additional file 1: Figure S1e, f). This RA-mediated induction of NTRK2 was sustained, and indeed continued to rise over longer RA treatments (Additional file 1: Figure S1e, f). While high NTRK2 expression combined with amplified MYCN is a marker for high-risk neuroblastoma [52], NTRK2 pathway activation by BDNF ligand treatment is also known to aid RA-mediated differentiation [53]. Our results indicate that RA dramatically upregulates NTRK2 expression, potentially priming the cells to respond to BDNF signalling.

### Transcriptome-wide profiling reveals novel regulatory mechanisms of known differentiation-associated genes

Of the genes regulated in opposing directions by RA and MYCN (Fig. 2a), we examined three in more detail: CYP26A1, LMO4 and ASCL1 (Fig. 2c). These genes were selected as they have previously been associated with either neuronal differentiation, MYCN or neuroblastoma, but our analysis reveals their opposing transcriptional regulation by RA and MYCN. Our RNA-seq analysis revealed that the expression of the CYP26A1 gene was massively increased upon RA treatment, jumping from almost undetectable to highly expressed (0.05–36.59 CPMkb; Fig. 2c). This increase was further enhanced by the combination of RA and Dox, despite Dox alone slightly reducing CYP26A1 expression (Fig. 2c). CYP26A1 is a member of the cytochrome P450 family and is involved in a negative feedback loop, where RA activates its expression while the CYP26A1 protein inactivates RA by hydroxylation [54–58]. CYP26A1 also regulates the production of migratory cranial neural crest cells [59]. Our data show a trend for MYCN overexpression to enhance the RA-induced expression of the RA inhibitor CYP26A1 (Fig. 2c).

LMO4 is a transcriptional regulator involved in the epithelial-to-mesenchymal transition of neuroblastoma and neural crest cells [60]. It can also inhibit differentiation of mammary epithelial cells and is overexpressed in breast cancer [61]. Its paralogue, LMO1, is a neuroblastoma oncogene which is duplicated in 12.4% of tumours, and is associated with aggressive disease [62]. LMO4 interferes with neuritogenesis in SY5Y cells [63], has a role in the differentiation of progenitor cells of motor neurons and the cranial neural crest and is highly expressed in proliferating mouse epithelial tissues [64, 65]. Our results reveal that LMO4 mRNA levels are upregulated by MYCN and downregulated by RA, while in the combination treatment MYCN overexpression partially reverses RA’s inhibitory effects on LMO4 expression (Fig. 2c).

The ASCL1 transcription factor stimulates neuronal differentiation, but its pro-differentiation functions are blocked by MYCN at the protein level, where MYCN maintains the phosphorylation of ASCL1 [9]. ASCL and MYCN also share some of the same promoter targets, but direct opposing regulation of these shared targets [66]. In addition to MYCN’s role in regulating phosphorylation of the ASCL1 protein, our data revealed that MYCN overexpression regulates ASCL1 mRNA levels (Fig. 2c). MYCN overexpression increased the level of ASCL1 mRNA, while RA treatment strongly reduced it (Fig. 2c). Combination treatment partially rescued the effect of RA on ASCL1. Therefore, ASCL1 is another gene differentially regulated by RA and MYCN overexpression, which is likely to contribute to MYCN’s ability to block neuronal differentiation.

In order to determine if the results obtained from the cell line were relevant to neuroblastoma tumour biology, we examined the effect of these genes on neuroblastoma patient survival in three large neuroblastoma tumour datasets (Versteeg [67], SEQC [68] and Kocak [69], with 88, 498 and 649 tumours, respectively), using the R2: Genomics Analysis and Visualization Platform (http://r2.amc.nl). CYP26A1, LMO4 and ASCL1 mRNA expression levels were each prognostic of patient outcome (Fig. 2d; Additional file 1: Figure S1g), consistent across the three datasets. Furthermore, the elevated expression of ASCL1 and LMO4 seen in the MYCN-overexpressing SY5Y-MYCN cells matched the high expression of these genes in the poor outcome tumours. Conversely, tumours with low ASCL1 and LMO4 expression had better prognosis, matching the cell line results in which these genes were downregulated by RA. The correlation between the RNA-seq and the tumour data was not as straightforward for CYP26A1. Expression of CYP26A1 was induced by RA and augmented further by MYCN induction, but not by MYCN induction alone (Fig. 2c). High levels of CYP26A1 were also indicative of poor outcome. Thus, while not activated by MYCN alone, CYP26A1 expression is induced by RA treatment even more strongly in the presence of elevated MYCN, and subsequently inactivates RA, resulting in retinoid resistance.

To move beyond the single-gene level and identify additional mechanisms through which MYCN overexpression can interfere with RA signalling we performed global pathway and network-based analysis of the RNA-seq data.

### Global analysis of mRNA-seq results reveals MYCN and RA differentially activate a range of transcriptional regulators

**Fig. 3:**
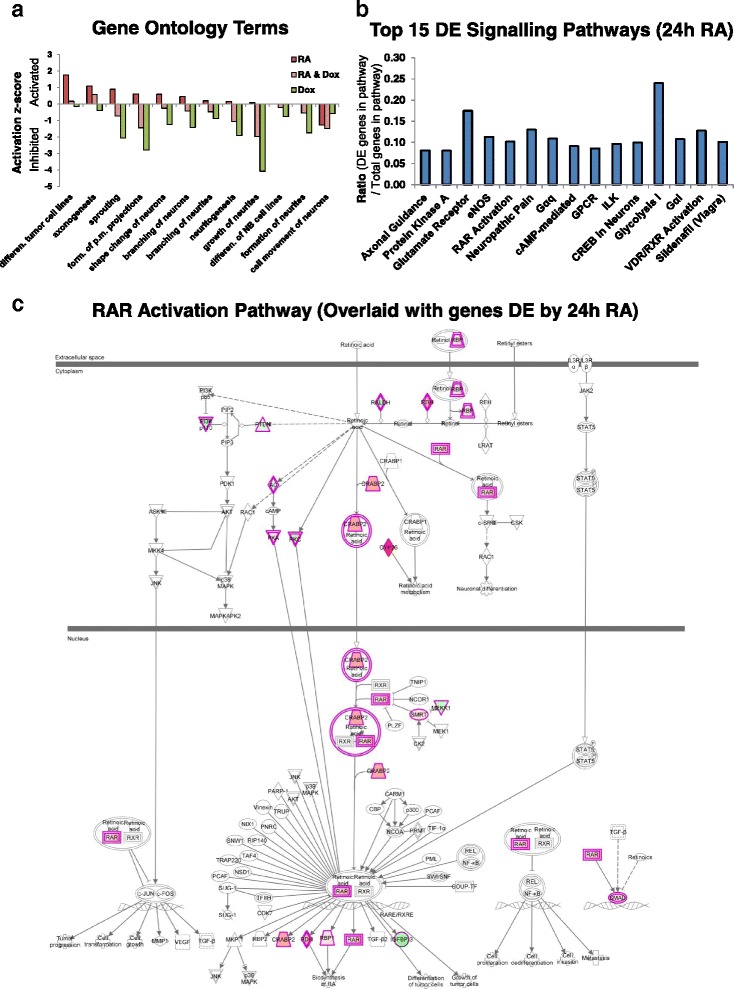
Gene ontology and signalling pathway analysis of the transcriptomic changes induced by RA and MYCN overexpression. **a** GO disease and function terms associated with neuronal differentiation, generated by analysing the SY5Y-MYCN RNA-seq data with IPA. GO term activation/inhibition levels are relative to those of the SY5Y-MYCN control cells. Full x-axis labels for the first, fourth and tenth bars, respectively, are: ‘differentiation of tumour cell lines’, ‘formation of plasma membrane projections’ and ‘differentiation of neuroblastoma cell lines’. **b** Top 15 differentially expressed signalling pathways (ranked by *p* value of overlap) between the 24-h 1-μM RA and 24-h 1-μl/ml DMSO vehicle control SY5Y-MYCN RNA-seq treatments, as generated by IPA analysis. **c** Retinoic acid receptor (*RAR*) signalling pathway overlaid with genes differentially expressed upon 24-h 1-μM RA treatment, as detected by RNA-seq. Pathway generated using IPA. *Red shaded genes* are upregulated by RA compared to control cells, while *green ones* are downregulated by RA. Intensity of shading indicates the degree of up- or downregulation, with deeper colours associated with higher levels of differential expression

We analysed the RNA-seq data using the GO disease and function terms tool of the IPA programme. Using existing knowledge, GO term analysis identifies patterns of gene regulation in the transcriptomic data which match patterns related to biological events such as apoptosis, ribosome biogenesis, proliferation and DNA replication. GO term analysis confirmed the phenotypic observations, showing that RA-activated genes are involved in neuronal differentiation processes and RA-inhibited genes are involved in cell movement (Fig. 3a). Conversely, MYCN overexpression repressed differentiation-associated processes, while combination treatment tended to fall between the two extremes but still with a bias towards the repression of neuronal differentiation (Fig. 3a). Disease and function GO analysis of the top 15 GO terms per condition revealed that RA inhibited proliferation and cancer-associated processes (Additional file 1: Figure S2a).

IPA analysis also showed that these phenotypic changes were achieved by RA differentially regulating the components of a number of signalling pathways associated with neuronal differentiation, including RAR and VDR/RXR, which were in the top 15 signalling pathways altered during differentiation (Fig. 3b). In particular, the expression of components of the RAR pathway itself were regulated by RA at all levels when projected onto a RAR pathway map (Fig. 3c). Aside from known RA-associated pathways, our analysis highlighted that a large array of signalling pathways participate in the differentiation of neurons, including axonal guidance, protein kinase A, eNOS and G-protein coupled receptor signalling (Fig. 3b).

**Fig. 4:**
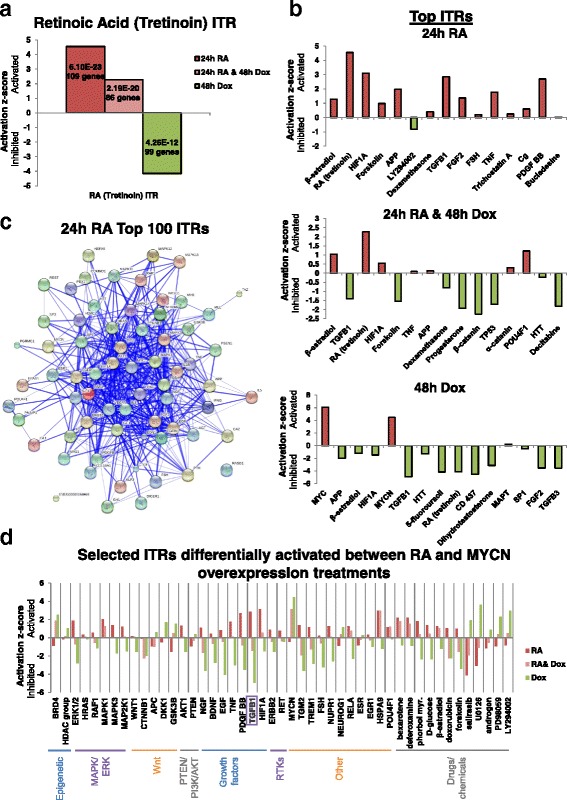
RA and MYCN overexpression drive opposing activation dynamics of their downstream transcriptional regulators. **a** Activation/inhibition score of the RA inferred transcriptional regulator (*ITR*) in SY5Y-MYCN cells across the treatment groups. These values are generated, using IPA, from the SY5Y-MYCN RNA-seq data. Activation/inhibition values are relative to the RA activity levels of the SY5Y-MYCN control cells. The total number of DE genes used to call the differential activity of the ITR and the *p* value of overlap are shown within each bar. **b** Top 15 ITRs (ranked by *p* value of overlap) from the SY5Y-MYCN RNA-seq data for each of the treatment groups compared with the DMSO vehicle control, as generated by IPA analysis. **c** Protein–protein interaction map of the top 100 ITRs governing the genes differentially expressed between the 24-h 1-μM RA and 24-h DMSO vehicle control treatments in SY5Y-MYCN cells. The protein interaction map of previously known connections between these proteins was generated with the String database. Note that only protein ITRs are shown; miRNAs and drug compounds are excluded. **d** Activation/inhibition scores of selected ITRs which were differentially activated between the RA and MYCN overexpression treatments. The scores were generated, using IPA, from the SY5Y-MYCN RNA-seq data. Activation/inhibition values are relative to the ITR activity levels of the SY5Y-MYCN control cells

The IPA suite was next used to identify the ITRs of DE genes of each treatment condition. Given the wealth of transcriptomic experiments publically available, a vast database exists regarding how genes are transcriptionally regulated in response to a wide array of regulators (genes, proteins or chemical compounds). ITR analysis harnesses this prior knowledge to identify patterns of transcriptional regulation in our datasets which match the patterns produced by known regulators. This comparison of known patterns versus patterns observed in the data enables the inference of which regulators are likely responsible for the differential gene expression seen in our transcriptomics data. Given this prior knowledge, ITR analysis can not only infer the regulators likely altering transcriptional regulation but also predict their activation status, i.e. whether these regulators were activated or inhibited in the treatment groups compared to the control cells [42, 70]. For more information on the statistical algorithms employed to match the detected changes in gene expression to known gene regulatory modules from the curated IPA knowledge database see Krämer et al. [70]. RA itself was a top ITR in each of the treatment groups (Fig. 4a, b). The analysis correctly and independently predicted it to be activated in both of the conditions in which RA treatment was performed (24-h RA and 24-h RA and 48-h Dox), providing a positive validation of the ITR analysis. As previously shown [42], RA as an ITR was inhibited by 48-h Dox treatment (Fig. 4a, b), revealing that MYCN DE regulates known RA target genes. Indeed, consistent with MYCN repressing RA’s effects on its target genes, the activation z score of RA for the combination treatment (24-h RA and 48-h Dox) was lower than for the 24-h RA single treatment, despite both conditions receiving the same dose and duration of RA treatment (Fig. 4a, b).

A clear trend emerged from the top ITRs of each condition: RA primarily activated transcriptional regulators (14/15) while MYCN primarily repressed them (12/15) (Fig. 4b). This trend was also clear across the top 100 ITRs (Additional file 1: Figure S3a; Additional file 6: Table S5). In line with this trend and the mutual antagonism of RA and MYCN, the combination treatment showed an almost equal number of ITRs to be activated and repressed, (seven and eight, respectively). The top protein ITRs of RA treatment formed a highly interconnected network, revealing the complexity of the molecular mechanisms involved in RA-mediated neuronal differentiation (Fig. 4c). Interestingly, almost half of the 24-h RA ITRs were chemical compounds, suggesting that additional drugs, if co-administered, may be able to improve the differentiation efficiency of clinical RA treatments.

### Antagonistic regulation of transcriptional regulators by RA and MYCN

To identify the transcriptional regulators through which MYCN exerts its inhibition of RA treatment, we next examined transcriptional regulators which were differentially activated between the treatment groups. A number of the top 15 ITRs were differentially regulated between the RA and MYCN overexpressing conditions, such as TGFB1, HIF1A, APP and FGF2 (Fig. 4b). To identify all ITRs which were differentially activated between RA and Dox treatments we overlapped the ITRs and their activation/inhibition status (Additional file 1: Figure S4a). Then, we generated protein interaction maps to reveal the transcriptional regulator networks which are likely to mediate MYCN inhibition of neuronal differentiation (Additional file 1: Figure S4a). The RA-inhibited and MYCN-activated protein ITRs were enriched for β-catenin binding genes (molecular function GO analysis, *p* = 8.52E-07) and Wnt signalling-related genes (KEGG pathway enrichment analysis, *p* = 4.84E-02), with all of the Wnt-related proteins present being antagonists of the pathway. Conversely, the protein–protein interaction network for the ITRs activated by RA and inhibited by MYCN overexpression were enriched for MAPK pathway-related proteins (KEGG pathway enrichment analysis, *p* = 6.829E-17). This network also included the WNT1 ITR, which combined with the results from the first network suggests that RA activates WNT1 signalling and represses Wnt antagonists. MYCN has the inverse effect, inhibiting WNT1 and activating Wnt antagonists. We have recently independently shown that Wnt and MAPK signalling are involved in regulating differentiation in MYCN-amplified neuroblastoma cells [42, 71]. In SY5Y cells, which are MYCN single-copy, the Wnt-RA cross-talk has been shown to be mediated by PSEN1 (Presenilin 1) [72]. We previously discovered novel cross-talk between the MYCN oncogene and GSK3 (one of the Wnt-related ITRs) [45], β-catenin [71] and MAPK [42]. Therefore, the protein–protein interaction network of regulators differentially activated by RA and MYCN overexpression identified here confirm our previous findings, support the validity of the current analysis and reveal that MYCN’s ability to inhibit RA-mediated differentiation involves the regulation of Wnt and MAPK signalling components.

To identify novel ITRs which may enhance the clinical response to RA when given as combination therapies, we next collated ITRs which were strongly differentially regulated between the three conditions (Fig. 4d). One of these regulators was MYCN itself. The effects of MYCN overexpression on known MYCN target genes predominated over RA effects, with MYCN overexpression strongly activating MYCN target gene expression, an effect which was only mildly attenuated by RA co-treatment (Fig. 4d). It should be noted, however, that this was in a MYCN-inducible system where MYCN expression was not under the control of its endogenous promoter and enhancers. Although artificial, this scenario mirrors highly amplified MYCN neuroblastoma where over 70 additional copies of the MYCN gene can be inserted in the tumour’s genome, often losing their endogenous promoters and enhancers. Interestingly, the strongly differentially regulated ITRs were significantly enriched for the Neurotrophin signalling pathway (BDNF, NGF and Trk receptors etc., see 'MYCN overexpression antagonises the normal transcriptional response to RA treatment' section above; KEGG *p* = 5.739E-10; Additional file 1: Figure S4b), which is strongly associated with neuronal differentiation and neuroblastoma outcome. These data suggest a convergence of the molecular mode of action of RA and neurotrophin (NGF/BDNF) mediated differentiation. The most highly connected nodes in the network included histone deacetylases (HDACs), which have recently been shown to synergise with RA treatment [8, 73–75], and TGFB1. TGFB1 is a ligand of the transforming growth factor beta (TGF-β) signalling pathway, with known roles in modulating differentiation [76–79]. The TGFB1 ITR was strongly differentially activated between RA and MYN overexpression conditions; RA activated TGFB1 while MYCN overexpression strongly inhibited it (Fig. 4d). The effect of MYCN was more dominant and TGFB1 activity was also inhibited in the co-treatment (Fig. 4d). We therefore further assessed the possibilities for TGFB1-informed therapy to enhance the response of MYCN-amplified cells to retinoid therapy.

### MYCN regulates TGFB1 and its target genes

**Fig. 5:**
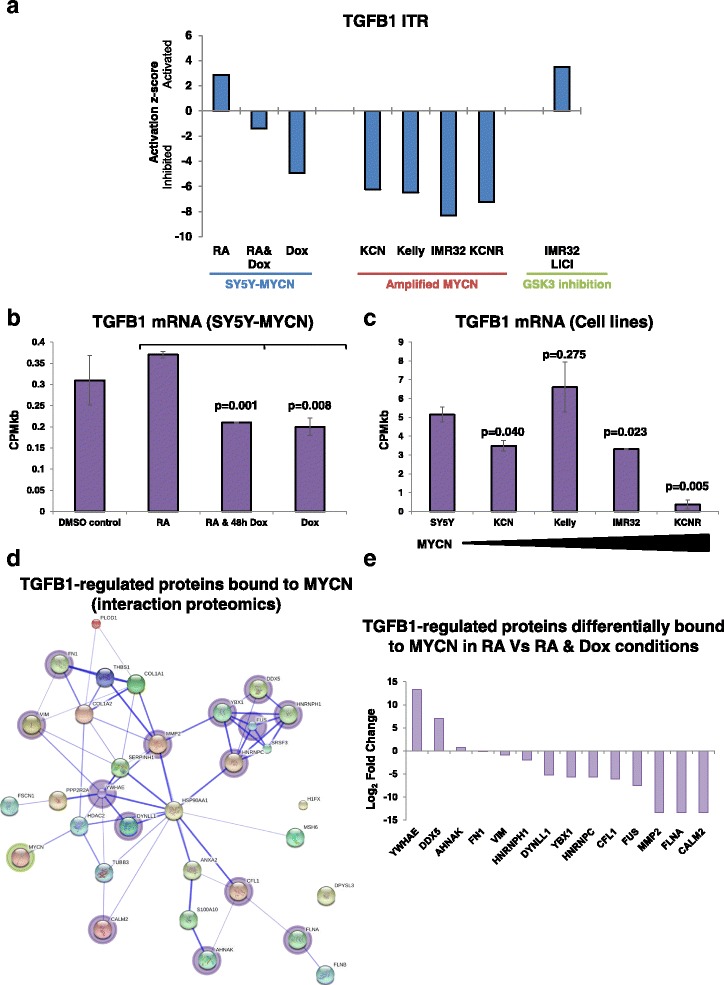
The TGFB1 transcriptional regulator is differentially activated by RA and MYCN overexpression and interacts with MYCN’s protein–protein interaction network. **a** Activation/inhibition score of the TGB1 ITR in neuroblastoma cells across a range of conditions: group 1, upon MYCN overexpression (SY5Y-MYCN cells); group 2, in amplified MYCN cell lines (*KCN*, *Kelly*, *IMR32* and *KCNR*) compared with MYCN single copy cells (SY5Y); group 3, IMR32 cells induced to undergo apoptosis by 24-h 28-mM LiCl treatment compared with control IMR32 cells. Values for each group are relative to the TGFB1 activity levels of the relevant control cells, SY5Y-MYCN un-induced, SY5Y cells and untreated IMR32 cells, respectively. These values were generated, using IPA, from RNA-seq data from both the present study and two of our previous publications [42, 45]. **b** Levels of absolute gene expression of TGFB1 mRNA in each of the SY5Y-MYCN RNA-seq treatments. Expression is in read counts per million adjusted by gene length in kilobases (*CPMkb*), with *error bars* denoting the standard deviation between replicates. *P* values above each bar are from *t*-test comparisons of TGFB1 expression in that condition with RA-treated cells. **c** Levels of absolute gene expression of TGFB1 mRNA across a panel of neuroblastoma cell lines. IMR32, Kelly, KCN and KCNR are lines with amplified MYCN, while SY5Y is a single copy MYCN cell line. Cell line data were mined from paired-end RNA-seq [42]. Expression is in read counts per million adjusted by gene length in kilobases (*CPMkb*), with *error bars* denoting the standard deviation between replicates. *P* values above each bar are from *t*-test comparisons of TGFB1 expression in that cell line with SY5Y cells. **d** Protein–protein interaction map of known TGFB1 targets (regulated proteins) which were found to bind to MYCN protein in the SY5Y-MYCN interaction proteomic dataset. The protein interaction map of previously known connections between these proteins was generated with the String database. **e** TGFB1 target proteins which showed differential binding (SY5Y-MYCN interaction proteomics) to MYCN protein when the RA-only treatment and the RA and Dox (MYCN overexpression) combination treatment were compared

To compare the effect of MYCN overexpression and RA on TGFB1 with the effects of amplified MYCN, we next examined sequencing datasets (RNA-seq and MYCN ChIP-seq) which we had previously generated (ArrayExpress (http://www.ebi.ac.uk/arrayexpress/) accession numbers E-MTAB-2690, E-MTAB-2691, E-MTAB-1684, E-MTAB-4100 and E-MTAB-2689) [42, 45, 71, 80]). These datasets revealed that TGFB1 was also a top regulator of the differences in the MYCN regulator and effector networks between single copy MYCN and MYCN-amplified neuroblastoma cell lines [42] (Fig. 5a). Mirroring the effect of MYCN overexpression in SY5Y-MYCN cells, the TGFB1 ITR was strongly inhibited in all MYCN-amplified cell lines compared with MYCN single copy cells. Our publicly available datasets also revealed that, similar to RA, induction of apoptosis by LiCl treatment (GSK3 inhibition) activated the TGFB1 ITR (Fig. 5a). Therefore, both cellular phenotypes associated with good outcome, i.e. differentiation and apoptosis, activated TGFB1 signalling.

We next examined MYCN ChIP-seq data [42] to determine if the inhibitory effect of MYCN on TGFB1 functioning (IPA ITR analysis) was achieved by MYCN binding to TGFB1 targets or binding to components of the TGF-β signalling pathway. Both overexpressed and amplified MYCN bound to the genes of a large number of TGFB1 targets (IPA ITR analysis), with the number of genes bound increasing with higher MYCN expression levels (Additional file 1: Figure S5a). Similarly, a proportion of the genes bound by MYCN have known SMAD regulatory elements (Additional file 1: Figure S5b), as revealed by DiRE analysis [81]. SMADs are the direct and prime transcriptional effectors of TGF-β signalling [82, 83]. In addition, genes bound by MYCN in KCNR cells were enriched for the TGF-β signalling pathway, as revealed by IPA (pathway analysis, *p*-value of overlap = 1.56E-02, ratio of overlap = 0.218), with 19 genes that encode components of the pathway being genomically bound by MYCN (Additional file 1: Figure S5c). TGF-β signalling pathway components were also bound by MYCN in KCN (17 genes) and 48-h Dox-induced SY5Y-MYCN (15 genes).

These results identified TGFB1 as a key regulator of RA-mediated differentiation, which is differentially activated in a MYCN context-dependent manner, being suppressed in MYCN-overexpressing and amplified cell lines. Therefore, we next examined if RA treatment and MYCN overexpression could modulate TGFB1 gene expression. MYCN overexpression modestly but significantly reduced TGFB1 mRNA levels compared to RA-only treated cells (*t*-tests, MYCN versus RA *p* value = 0.0076, RA versus co-treatment [MYCN and RA] *p* value = 0.0010; Fig. 5b). In line with the MYCN overexpression results, three of the four MYCN-amplified cell lines had lower TGFB1 mRNA expression than SY5Y cells which are MYCN single-copy, with expression of TGFB1 in KCNR cells being almost absent (Fig. 5c). The inhibitory effect of MYCN upon TGFB1 mRNA expression was not rescued by RA treatment (Fig. 5b), suggesting a novel mechanism through which MYCN can inhibit RA-mediated neuronal differentiation.

While RA did not rescue the effect of MYCN overexpression on TGFB1 mRNA levels, it partially rescued TGFB1 signalling, as revealed by the ITR analysis (Fig. 5a). Additionally, the inhibitory effects of MYCN on TGFB1 functioning in the other cell lines (Fig. 5a) did not always correlate directly to TGFB1 mRNA expression levels (Fig. 5c), suggesting further levels of cross-talk. Therefore, to further probe the functional relationship between TFGB1 and MYCN we next examined MYCN’s protein–protein interactions.

### MYCN protein interacts with TGF-β signalling-associated proteins

While RA slightly increased TGFB1 mRNA levels and MYCN overexpression reduced them, these changes do not fully account for the differences in TGFB1 activation revealed by the ITR analysis. Therefore, to investigate additional potential cross-talk between MYCN and TGF-β signalling we performed MYCN interaction proteomics using the same experimental conditions as for the RNA-seq experiments: (i) 24-h DMSO (control); (ii) 24-h RA; (iii) 24-h RA and 48-h Dox; and (iv) 48-h Dox. We performed IPA ITR analysis on the proteins bound by MYCN in all conditions, which revealed that 32 TGFB1-regulated proteins had protein–protein interactions with MYCN (Fig. 5d). Of these, only MYCN’s interaction with HDAC2 was previously known (present in the String database). Fourteen of the 32 proteins were differentially bound to MYCN when RA-only treatment was compared with the RA and Dox co-treatment, with the majority of them binding less strongly to MYCN in the MYCN overexpressing sample (Fig. 5e). For 13 of the 14 differentially bound proteins, the change in MYCN binding was not as a result of altered transcriptional regulation (Additional file 1: Figure S5d); rather, altered binding was likely mediated by post-translational events. Interestingly, the one protein in which altered transcriptional regulation may have contributed to its differential binding to MYCN protein, at least partially, was the neuroblast differentiation-associated protein AHNAK. AHNAK was differentially expressed at the mRNA level, with opposing regulation by MYCN overexpression and RA treatment (Fig. 2a; Additional file 1: Figure S5d). AHNAK was bound to MYCN in all conditions, and has been described as a tumour suppressor that can stimulate the growth suppressing functions of the TGF-β pathway [84].

Taken together, our findings reveal cross-talk between MYCN and the TGF-β pathway at several levels, including the regulation of TGFB1 mRNA expression, the regulation of TGFB1 target genes and pathway components, and protein–protein interactions between MYCN- and TGFB1-regulated gene products. They also show that MYCN and RA drive opposing functional regulation of TGFB1.

The ability of MYCN overexpression to inhibit the normal RA-mediated activation of TGFB1 signalling, revealed here, further underscores the importance of TGF-β suppression in contributing to MYCN’s oncogenic potential. We therefore further investigated associations of TGF-β signalling genes and outcome in neuroblastoma tumour data and whether pharmaceutical modulation of the TGF-β pathway can enhance the effectiveness of RA treatment in MYCN-amplified cell lines.

### Genes mediating TGF-β signalling activation in response to the small molecule kartogenin are prognostic of neuroblastoma patient outcome

**Fig. 6:**
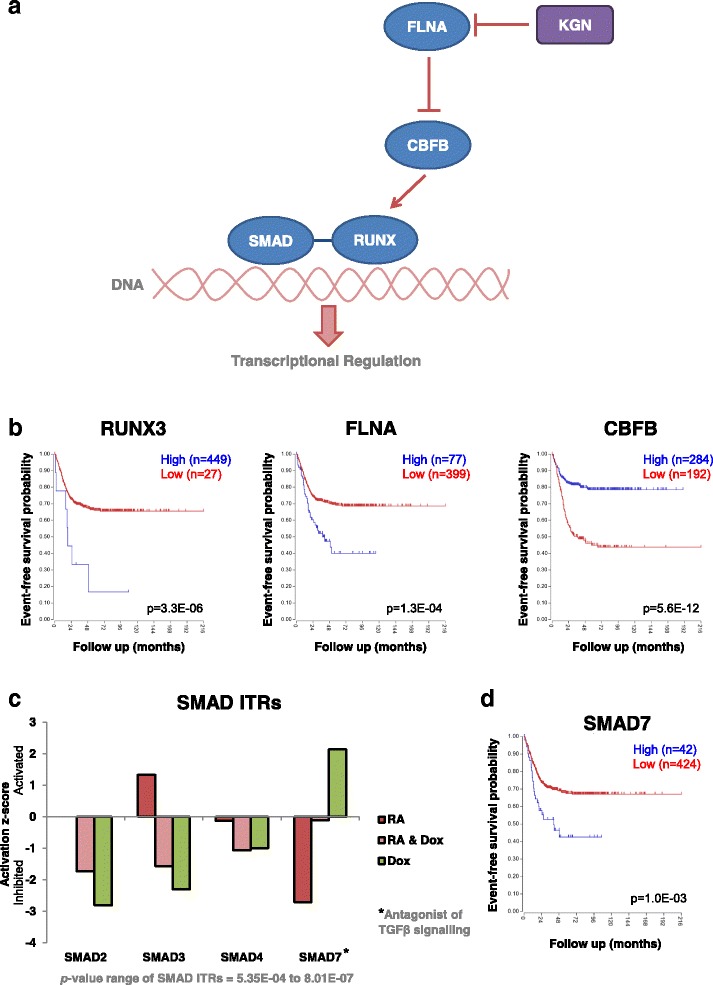
The components of the TGF-β pathway targeted by the small molecule kartogenin are predictive of neuroblastoma patient outcome. **a** Schematic of the SMAD-RUNX portion of the TGF-β signalling pathway, showing how it is targeted by kartogenin (*KGN*). KGN inhibits FLNA, a negative regulator of the pathway, thereby activating SMAD-RUNX-dependent transcriptional regulation. This pathway is a modified version of the TGF-β pathway described in IPA. **b** Kaplan–Meier survival curves showing the predictive strength of the expression levels of the RUNX3, FLNA and CBFP mRNAs in neuroblastoma tumours on patient outcome. Plots generated using the Kocak [69] 649 neuroblastoma tumour dataset in the R2: Genomics Analysis and Visualization Platform (http://r2.amc.nl). **c** Activation/inhibition score of the SMAD ITRs which were altered in SY5Y-MYCN cells across the treatment groups. These values are generated, using IPA, from the SY5Y-MYCN RNA-seq data. Activation/inhibition values are relative to the corresponding SMAD activity levels of the SY5Y-MYCN control cells. **d** Kaplan–Meier survival curves showing the predictive strength of SMAD7 expression levels in neuroblastoma tumours on patient outcome. Plots generated as described for panel **b**

Having identified a role for TGF-β signalling in MYCN’s blocking of RA-mediated neuronal differentiation, we next assessed whether this shared node of MYCN and RA signalling was amenable to therapeutic intervention. Kartogenin (KGN) is a recently developed small molecule which enhances the activation of TGF-β signalling through indirectly regulating the activity of the SMAD transcriptional effectors [85, 86]. KGN has been shown to promote chondrocyte differentiation in vitro and in vivo [85–87]. KGN also strongly upregulates the expression of TGFB1 itself [87]. KGN competitively binds filamin A (FLNA), thus inhibiting it from interacting with core-binding factor β subunit (CBFB) [85]. CBFB that is not bound by FLNA is then free to translocate from the cytoplasm to the nucleus, where it complexes with the RUNX-SMAD transcriptional machinery and regulates target gene expression (Fig. 6a). Interestingly, we showed that SMADs 1, 2 and 6 and RUNX2 were genomically bound by amplified MYCN (Additional file 1: Figure S5c) and there was a protein–protein interaction between MYCN and FLNA (Fig. 5d, e), revealing further levels of cross-talk between TGF-β signalling, KGN’s mode of action and oncogenic MYCN. The RUNX developmental regulators have previously been implicated in a variety of cancers [88]. Therefore, to examine whether the FLNA-CBFB-RUNX2-SMADs module is associated with neuroblastoma tumour biology and patient outcome, we examined the R2 neuroblastoma datasets (used above; http://r2.amc.nl) for these genes. Low CBFB and high FLNA (inhibitor of CBFB) mRNA expression was associated with poor patient outcomes (Fig. 6b; Additional file 1: Figure S6a, b). In line with the differential activation of the TGFB1 ITR by RA and MYCN, the SMAD transcriptional effectors of the pathway were also largely differentially activated between the two conditions, with the canonical SMADs being inhibited by MYCN overexpression (Fig. 6c). Interestingly, SMAD7, an antagonist of TGF-β signalling, was activated by MYCN and inhibited by RA, potentially revealing another mechanism through which MYCN inhibits RA-mediated activation of TGFB signalling. Accordingly, high SMAD7 mRNA expression was predictive of poor patient outcomes (Fig. 6d). Additionally, a number of the other SMAD genes were predictive of patient outcome, with high SMAD1 and SMAD2 expression associated with good outcomes (Additional file 1: Figure S6c), while RUNX3 showed a better survival prediction than RUNX2 (Fig. 6b; Additional file 1: Figure S6a–c).

### KGN shows differentiating potential for neuroblastoma but does not overcome the amplified-MYCN-mediated differentiation block

**Fig. 7:**
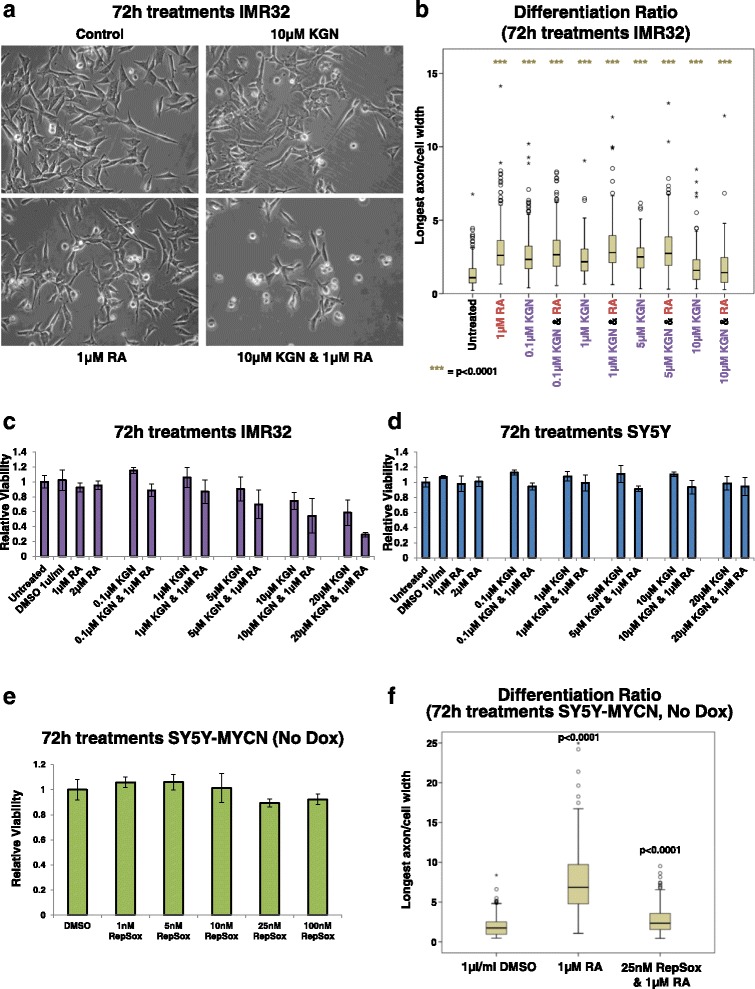
Kartogenin and retinoic acid cooperate to reduce the viability of MYCN-amplified neuroblastoma cells. **a** Imaging of IMR32 cells treated for 3 days singly with 1 μM RA or 10 μM KGN, or a combination of both. All panels are imaged at 40× magnification. **b** The differentiation ratio of IMR32 cells treated for 3 days with individual agents (KGN or RA) or combination treatments (KGN and RA). The images from three biological replicates were pooled and then measurements of individual cells were made using ImageJ v1.44p (http://imagej.nih.gov/ij). The range of measured cells (N) per treatment group is 177–352. The differentiation ratio of each cell was calculated by dividing the length of its longest axon by the cell’s width. All treatment groups are significantly different (with a *p* value <0.0001) to the untreated control cells. **c**, **d** Cell viability analysis of MYCN-amplified IMR32 (**c**) or single-copy MYCN parental SY5Y (**d**) cells treated for 72 h with KGN and RA, either individually or in combination, as detected by MTS assay. Viability is set relative to that of the respective control untreated cells (IMR32 or SY5Y), with *error bars* denoting the standard deviation between replicates. **e** Cell viability analysis of un-induced SY5Y-MYCN cells treated for 72 h with the TGF-βR1 inhibitor RepSox, as detected by MTS assay. Viability is set relative to that of the respective DMSO vehicle-control cells, with *error bars* denoting the standard deviation between replicates. **f** The differentiation ratio of un-induced (no Dox) SY5Y-MYCN cells treated for 3 days with RA-only or RA and RepSox combination treatment. The images from three biological replicates were pooled and then measurements of individual cells were made using ImageJ v1.44p (http://imagej.nih.gov/ij). The range of measured cells (N) per treatment group is 135–151. The differentiation ratio of each cell was calculated by dividing the length of its longest axon by the cell’s width. *P* values (*t*-test) of each treatment group compared with the DMSO-vehicle controls are shown above each box

Having demonstrated cross-talk between MYCN and the TGF-β signalling pathway, and having shown that pathway components targeted by KGN are associated with neuroblastoma patient outcome, we next treated MYCN-amplified IMR32 cells to determine their response to KGN individually and in combination with RA. IMR32 cells are less responsive to RA-mediated differentiation than MYCN single-copy cells. Low dose KGN (0.1–5 μM) was almost as effective at differentiating IMR32 cells as RA (Fig. 7a, b), with all treatment groups being significantly different to untreated controls (*p* < 0.0001 for all groups versus controls). It should be noted that MYCN-amplified IMR32 cells are far more resistant to differentiation than MYCN single-copy neuroblastoma cells (see Fig. 1a for comparison). Interestingly, when given in combination with RA, none of the low dose KGN (0.1–5 μM) treatments enhanced the differentiating potential over that seen in RA-only treatments (*t*-test, *p* = 0.226–0.982). High dose KGN (10 μM) had a significantly different differentiation ratio to untreated cells (*t*-test, *p* < 0.0001), but the extent of differentiation was less than that seen in lower dose KGN treatments (0.1–5 μM KGN). It was also able to partially inhibit the differentiating effects of RA in the combination treatment compared with RA-only treatment (*t*-test, *p* < 0.0001).

While both RA and KGN increased the differentiation ratio of IMR32 cells, the combination of these compounds did not act synergistically to overcome the differentiation block imposed by amplified MYCN (Fig. 7a, b). However, combination treatment of RA with a high dose of KGN (10 μM) resulted in a large proportion of cells with an apoptotic-like rounded appearance (Fig. 7a; Additional file 1: Figure S7a). These apoptotic-like cells were not included in the calculation of the differentiation ratios due to their complete lack of axons; only surviving cells were measured. In order to further assess the effect of KGN on neuroblastoma cells, we next assessed cell viability.

### Combination treatment of KGN and RA specifically reduces viability of MYCN-amplified cells, but not MYCN single-copy cells

The viability of IMR32 cells was reduced in a dose-dependent manner by KGN (Fig. 7c), though the effect was relatively mild. Our analysis suggested that combination treatment with these two compounds should provide an additive or synergistic effect. Despite RA having no effect on cell viability, RA treatment sensitised IMR32 cells to KGN, with co-treated cells responding more strongly than to each compound individually (Fig. 7a, c). Combination treatment of 20 μM KGN and 1 μM RA strongly reduced cell viability (*t*-test, *p* = 1.0E-04; Fig. 7c). Combination treatment significantly reduced cell viability compared with 20 μM KGN-only treatment (*t*-test, *p* = 8.4E-03). MYCN-amplified KCNR cells also showed a strong reduction in cell viability upon combination treatment (Additional file 1: Figure S7b; *t*-test, *p* = 4.0E-03). Importantly, in line with the omics analysis, KGN-RA combination treatments only reduced the viability of MYCN-amplified cells, without significant effects on the viability of the MYCN single-copy cell line SY5Y (untreated versus 20 μM KGN and 1 μM RA; *t*-test, *p* = 0.4715; Fig. 7d). Combination treatment also significantly reduced the cell viability of NBL-S cells (Additional file 1: Figure S7c; untreated versus 20 μM KGN and 1 μM RA; *t*-test, *p* = 1.0E-04), which although being MYCN single copy have elevated levels of MYCN protein due to an increased MYCN protein half-life [89].

While KGN and RA did not cooperate to further differentiate IMR32 cells, they did cooperate to enhance the apoptotic response to treatment. As part of the RA neuronal differentiation programme, RA is known to block proliferation and promote apoptosis of normal neuronal precursors and low-risk neuroblastoma cells, in addition to inducing the differentiating of surviving cells [90–94]. Similarly, ITR analysis revealed that TGFB1 was strongly activated when IMR32 cells were induced to undergo apoptosis by LiCl treatment (Fig. 5a). To further functionally confirm that TGFB1 is involved in directing neuroblastoma cell fate in a MYCN-dependant manner, we mined an RNAi knockdown screen targeting the druggable genome in SY5Y-MYCN cells [42]. TGFB1 was a top ITR (ranked third) of the 674 genes that strongly reduced SY5Y-MYCN cell viability when knocked down (Additional file 1: Figure S7d), confirming the functional role of TGFB1 in neuroblastoma cell fate and supporting its likely therapeutic potential. RA was also a high ranking (12th) ITR of these viability-reducing genes (Additional file 1: Figure S7d).

While elevated MYCN levels can block the pro-apoptotic effects of RA (Additional file 1: Figure S7e), our results reveal that KGN can be used as a combination therapy to promote MYCN-amplified neuroblastoma cell death. Thus, RA and KGN combination treatments represent a novel therapeutic option with potential for targeting high-risk MYCN-amplified tumours.

### Pharmacological inhibition of TGF-β signalling strongly attenuates RA-mediated neuronal differentiation

To confirm the functional role of the MYCN-induced inhibition of TGF-β signalling in promoting retinoid resistance we next investigated whether pharmacological inhibition of TGF-β signalling could attenuate RA-mediated differentiation in the absence of MYCN overexpression. We treated SY5Y-MYCN cells with RepSox, a potent and selective inhibitor of TGF-β receptor 1 (TGFBR1) [95]. RepSox can successfully replace Sox2 in reprogramming cells and its use alongside other reprogramming factors is efficient at generating induced pluripotent stem cells [96]. RepSox did not reduce the viability of SY5Y-MYCN cells (Fig. 7e; DMSO-control versus 100 nM RepSox; t-test, *p* value = 0.2244). When un-induced SY5Y-MYCN cells were treated with RA and RepSox, RepSox blocked the differentiating effect of RA so strongly (1 μM RA-only versus 25 nM RepSox and 1 μM RA; t-test, *p* value <0.0001.0E-04) as to maintain the differentiation ratio near the same level as that seen in control cells (Fig. 7f). Taken together, our results reveal that TGF-β signalling inhibition, whether achieved by MYCN overexpression or pharmacological treatment, strongly contributes to resistance to retinoid-mediated differentiation in neuroblastoma cells and that pharmacological activation of TGF-β signalling represents a promising strategy to sensitising MYCN-amplified cells to retinoid-mediated apoptosis.

## Discussion

Retinoids are an important component of advanced neuroblastoma therapy, yet half of all patients treated with isotretinoin (13-cis retinoic acid) still relapse and die [97]. Therefore, more effective combination therapies, with a lower side effect profile, are required to improve outcomes [97]. Our omic data enhance our understanding of RA and MYCN’s antagonistic relationship, revealing the molecular mechanisms through which MYCN inhibits normal RA-mediated neuronal differentiation and suggesting novel combination therapies. The gene-level analysis successfully identified targets known to be involved in retinoid-induced neuroblastoma differentiation, which bolsters confidence in the novel genes identified. Known genes identified include ASCL1, RET, LMO4, CYP26A1, DKK2 and ODC1 [8, 9, 63, 98–103], many of which have also previously been identified as MYCN target genes whose expression levels are correlated to patient survival outcome. Here we show that these genes are differentially regulated by RA and MYCN, with MYCN overexpression preventing their normal transcriptional response to RA in the co-treatment condition.

The global analysis revealed that many transcriptional regulators had differential activity (activation/inhibition) induced by RA and MYCN overexpression. These regulators included expected differentiation-associated ligands and receptors such as NGF, BDNF, PDGF, RET [52, 104–106] and RA itself, which provides further confirmation of our analysis approach. Additionally, three regulators, Wnt, β-estradiol and MAPK, which we recently independently identified as being key components of the amplified-MYCN signalling network [42, 71] with a role in neuroblastoma differentiation, were shown here to be differentially activated by RA and MYCN overexpression. We recently demonstrated that targeting each of these pathways altered MYCN-amplified neuroblastoma viability and differentiation and that co-treatment with RA had additive or synergistic effects [42, 71], suggesting that such combination therapies could be useful to treat MYCN-amplified neuroblastoma. MAPK (MEK) has also previously been linked to MYCN-independent NF1-mediated RA resistance, and MEK inhibition combined with RA treatment was proposed as a potential strategy to treat NF1-deficient neuroblastomas [36]. MAPK inhibition and RA co-treatment has recently been confirmed to synergistically reduce neuroblastoma tumour growth in vivo using zebrafish models [107]. Similar to other genes identified in our analysis, the neurotrophin receptor TrkB (NTRK2) and the Wnt, β-estradiol and MAPK signalling pathways have all been shown to have a strong correlation with poor prognosis in neuroblastoma [42, 71, 108, 109]. Furthermore, MAPK and Wnt-associated genes (including LEF1) have been shown to be recurrently mutated in relapsed neuroblastoma [108, 110]. The emergence of the above molecules and pathways in the present RA-mediated neuroblastoma differentiation study further confirms their therapeutic potential.

By studying downstream signalling through the combination of transcriptomics and computational-inference of driving regulators, rather than genetic triggers (such as mutations and epigenetic modifications), our approach can identify a large range of relevant transcriptional regulators, including epigenetic ones. Our approach provides a powerful tool for precision oncology by identifying functional changes in signalling networks that drive malignancies but may be the result of different or combinatorial (epi)genetic events that are often difficult to interpret in their own right not primarily driven by somatic mutations, such as [38]. Given the paucity of somatic mutations in paediatric cancers, including neuroblastoma [3, 38], it is likely that altered epigenetic regulation can contribute to the widespread resistance to retinoid therapy. For instance, it has been shown that epigenetic regulation of the RARA gene (promoter methylation) can block the subsequent differentiation of neuronal precursor cells [37]. Indeed, MYCN is known to interact with the epigenetic machinery [111, 112]. Our results revealed differentially activated epigenetic regulators, including HDACs and BRD4, thus confirming the recently identified role for HDACs [8, 73–75] in neuroblastoma responsiveness to RA. Retinoids were also the top hits in a small molecule library screen for compounds which enhanced HDAC inhibitor-mediated neuroblastoma differentiation in vitro and xenograft regression in vivo [74]. Consistent with these results, our data reveal that RA inhibits HDAC functioning while MYCN overexpression activates it.

It is interesting to note that RA itself was a top ITR of the MYCN interaction partners in all three conditions (RA only, Dox only, and RA and Dox). RA was a top ITR when all of the MYCN-bound proteins were considered (*p* value of overlap range 1.23E-16 to 1.81E-16), but also when only those proteins were considered which were differentially bound between conditions (*p* value of overlap range 4.28E-11 to 3.86E-12). These results suggest that in addition to altering transcription of MYCN, RA transcriptionally regulates a large number of MYCN’s interacting proteins, potentially re-wiring MYCN’s interaction network.

A key differentially activated regulator was TGFB1, a ligand of the TGF-β signalling pathway. We identified MYCN-RA-TGF-β cross-talk at several molecular levels, including MYCN’s genomic binding, transcriptional activities and protein–protein interactome. TGFB1 was previously shown to be induced by RA in RA-responsive neuroblastoma cell lines [113]. Our data show that TGFB1’s effect as a transcriptional regulator was strongly inhibited by MYCN overexpression or amplification and strongly activated by RA. Interestingly, MYCN’s effect on TGFB1 activity was dominant, with TGFB1 being repressed in RA treated cells with MYCN overexpression. We found that MYCN overexpression alters TGFB1 functioning, not just by transcriptionally regulating TGFB1 mRNA but also through protein–protein interactions with a number of TGF-β signalling-associated proteins.

TGF-β signalling has previously been linked to adult neurogenesis [114, 115], FGF2-mediated neuroblastoma differentiation [116], and neuroblastoma cell invasiveness [117]. While TGF-β can promote tumour progression in a number of adult cancers [118, 119], we show that in neuroblastoma TGF-β is inhibited by MYCN overexpression and MYCN amplification. We also show that TGF-β inhibition, whether achieved through elevated MYCN levels or pharmacological means, strongly contributes to retinoid resistance. Additionally, the miR-17-92 microRNA cluster has been shown to downregulate TGF-β signalling in neuroblastoma and is a marker for poor survival [120]. Similarly, TGF-β receptor (type III) is reduced in high-stage neuroblastoma, while increased TGF-β receptor (type II) expression suppresses malignant neuroblastoma phenotypes and induces differentiation [121, 122]. Our findings combined with the known associations between TGF-β signalling and neuronal differentiation prompted us to investigate the therapeutic potential of pharmaceutically activating TGF-β signalling in neuroblastoma using KGN. As neuroblastoma cells can be induced to differentiate even in the presence of a certain level of MYCN overexpression [31], we investigated KGN-RA combination treatments in the MYCN-amplified and RA-resistant IMR32 cell line. While both compounds produced a modest increase in the differentiation ratio of IMR32 cells, the combination treatment primarily reduced the viability of MYCN-amplified cells (IMR32 and KCNR cell lines). This therapeutic combination therefore demonstrates potential for the treatment of high-risk MYCN-amplified patients. We also show that KGN has differentiating potential in a neuronal lineage. Our results suggest that KGN’s effectiveness in promoting neuronal stem cells to differentiate should be investigated further, particularly its effects in normal non-oncogenic MYCN single-copy neuroblast cells. KGN may have potential for neuronal regenerative medicine, similar to its proposed uses for manipulating bone marrow stromal cells, mesenchymal stem cells and patellar tendon stem cells for tissue repair and regeneration [85, 86]. However, unlike in bone marrow stromal cells and patellar tendon stem cells where KGN also increases proliferation, KGN induced a loss of viability in MYCN-amplified neuroblastoma cells, showing therapeutic potential, an effect which was further enhanced by co-treatment with RA. KGN is a first-generation TGF-β signalling activator, with its ability to promote mesenchymal stem cells into chondrocytes being identified through an image-based high-throughput screen [85]. Therefore, in addition to further pre-clinical studies of RA-KGN combinations, it will be important to evaluate the effectiveness of future iterations of small molecule TGF-β signalling activators as potential neuroblastoma therapeutics and enhancers of neuronal differentiation.

## Conclusions

By applying genomic-level omics approaches to the signalling networks regulating neuroblastoma differentiation and stemness, we have determined the regulators involved in the MYCN-mediated inhibition of neuroblastoma cell differentiation. We have revealed a large network of interconnected regulators governing neuronal differentiation, which are differentially regulated by the pro-differentiation compound RA and by differentiation inhibiting MYCN overexpression. A key differentially regulated pathway was TGF-β signalling. TGF-β inhibition (RepSox) was sufficient to strongly attenuate RA-mediated differentiation even in the absence of MYCN overexpression. We have shown that co-targeting of the retinoic acid and TGF-β pathways, through RA and KGN (a small molecule TGF-β activator) combination treatment, strongly reduces MYCN-amplified RA-resistant neuroblastoma cell viability.

## Additional files

Additional file 1: Figure S1. Additional RT-qPCR, RNA-seq and patient survival curve data. **Figure S2** Additional GO term analysis for the SY5Y-MYCN RNA-seq data. **Figure S3** Additional ITR analysis for the SY5Y-MYCN RNA-seq data. **Figure S4** Additional data showing that RA and MYCN overexpression drive opposing activation dynamics of their downstream transcriptional regulators. **Figure S5** Additional TGF-β/SMAD MYCN ChIP-seq and interactome data. **Figure S6** Additional data showing TGF-β pathway components targeted by the small molecule kartogenin are predictive of neuroblastoma patient outcome. **Figure S7** Additional kartogenin and retinoic acid cell viability and apoptosis data. (PDF 10467 kb)
Additional file 2: Table S1. CPMkb expression values for all genes in each RNA-seq sample. (XLSX 5902 kb)
Additional file 3: Table S2. Genes differentially expressed by 24-h RA treatment. (XLSX 73 kb)
Additional file 4: Table S3. Genes differentially expressed by 24-h RA and 48-h Dox co-treatment. (XLSX 54 kb)
Additional file 5: Table S4. Genes differentially expressed by 48-h Dox (MYCN overexpression) treatment. (XLSX 86 kb)
Additional file 6: Table S5. Top 100 ITRs per treatment, as identified by IPA from the RNA-seq data. (XLSX 100 kb)

## Abbreviations

ATRA: All-trans-retinoic acid
ChIP: Chromatin immunoprecipitation
CPMkb: Read counts per million adjusted by gene length in kilobases
DE: Differentially expressed
Dox: Doxycycline
GO: Gene ontology
HDAC: Histone deacetylase
IPA: Ingenuity Pathway Analysis
ITR: Inferred transcriptional regulator
KGN: Kartogenin
RA: Retinoic acid
RAR: Retinoic acid receptor
RNA-seq: RNA sequencing
RT-qPCR: Quantitative RT-PCR
TGF-β: Transforming growth factor beta
