# Supplementary material for: Retinoic acid and TGF-β signalling cooperate to overcome MYCN-induced retinoid resistance
Source: Genome Med. 2017 Feb 10;9:15. doi: 10.1186/s13073-017-0407-3 (PMC5303304; doi:10.1186/s13073-017-0407-3)
Supplement: Additional file 1: Figure S1. — Additional RT-qPCR, RNA-seq and patient survival curve data. Figure S2 Additional GO term analysis for the SY5Y-MYCN RNA-seq data. Figure S3 Additional ITR analysis for the SY5Y-MYCN RNA-seq data. Figure S4 Additional data showing that RA and MYCN overexpression drive opposing activation dynamics of their downstream transcriptional regulators. Figure S5 Additional TGF-β/SMAD MYCN ChIP-seq and interactome data. Figure S6 Additional data showing TGF-β pathway components targeted by the small molecule kartogenin are predictive of neuroblastoma patient outcome. Figure S7 Additional kartogenin and retinoic acid cell viability and apoptosis data. (PDF 10467 kb) [file 13073_2017_407_MOESM1_ESM.pdf]

Figure S1

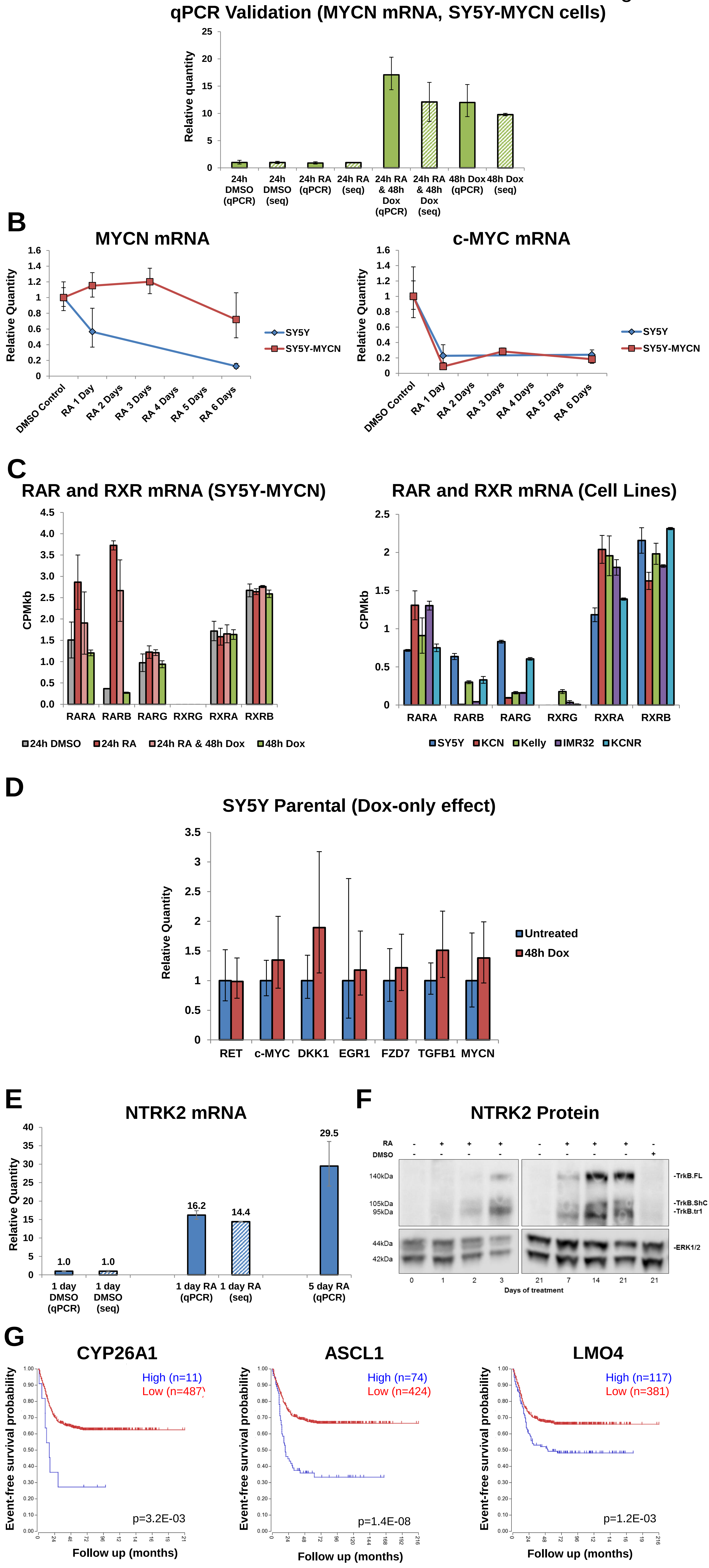

Figure S1. Additional RT-qPCR, RNA-seq and patient survival curve data. (A) RT-qPCR validation of the RNA-seq data showing the differential expression of MYCN between treatment groups. Levels of MYCN expression are set relative to those in untreated samples of control cells. Time-points mirror the RNA-seq time-points. RT-qPCR samples denoted by (qPCR) with their RNA-seq counterparts denoted by (seq). Error bars for qPCR samples denote RQ Min and RQ Max, while for RNA-seq samples they are standard deviation. **(B)** RT-qPCRs of the relative quantity of MYC gene expression in response to a 1µM RA time-course treatment, MYCN (left) and c-MYC (right) in the parental SY5Y cell line and in SY5Y-MYCN cells. MYCN expression in the SY5Y-MYCN cell line is under the control of a tetracycline inducible promotor and not its endogenous promotor, and the transcript lacks its untranslated regions. Levels of expression are set relative to those in untreated samples of the respective cell lines. Error bars denote RQ Min and RQ Max values. **(C)** Levels of absolute gene expression of the retinoic acid receptor genes in each of the SY5Y-MYCN RNA-seq treatments (left) and the neuroblastoma cell line RNA-seq [42] (right). Expression is in read counts per million adjusted by gene length in kilobases (CPMkb), with error bars denoting the standard deviation between replicates. **(D)** RT-qPCR of the effects of 48h Dox treatment in SY5Y parental cells on the expression of genes DE regulated by MYCN overexpression in SY5Y-MYCN cells (Figs 2B, 5B, S1A). SY5Y cells lack the Dox-inducible MYCN overexpression of SY5Y-MYCN cells, therefore any Dox-mediated changes in gene expression in SY5Y parental cells are solely the result of direct Dox effects. Error bars denote RQ Min and RQ Max. **(E)** RT-qPCR validation of the RNA-seq for the differentially expressed gene NTRK2 (TrkB). Levels of expression are set relative to those in untreated samples of control cells. Time-points mirror the RNA-seq time-points, with an additional qPCR time-point of 5 days. RT-qPCR samples denoted by (qPCR) with their RNA-seq counterparts denoted by (seq). Error bars for qPCR samples denote RQ Min and RQ Max, while for RNA-seq samples they are standard deviation. **(F)** Western blot showing changes in TrkB (NTRK2) protein (full length [FL] and truncated isoforms [ShC and tr1]) in response to 1µM RA treatment in the SY5Y-MYCN cell line. **(G)** Kaplan-Meier survival curves showing the predictive strength of the expression levels of the CYP26A1, ASCL1 and LMO4 mRNAs in neuroblastoma tumours on patient outcome. Curves generated using the SEQC [68] 498 neuroblastoma tumour dataset in the R2: Genomics Analysis and Visualization Platform (http://r2.amc.nl).

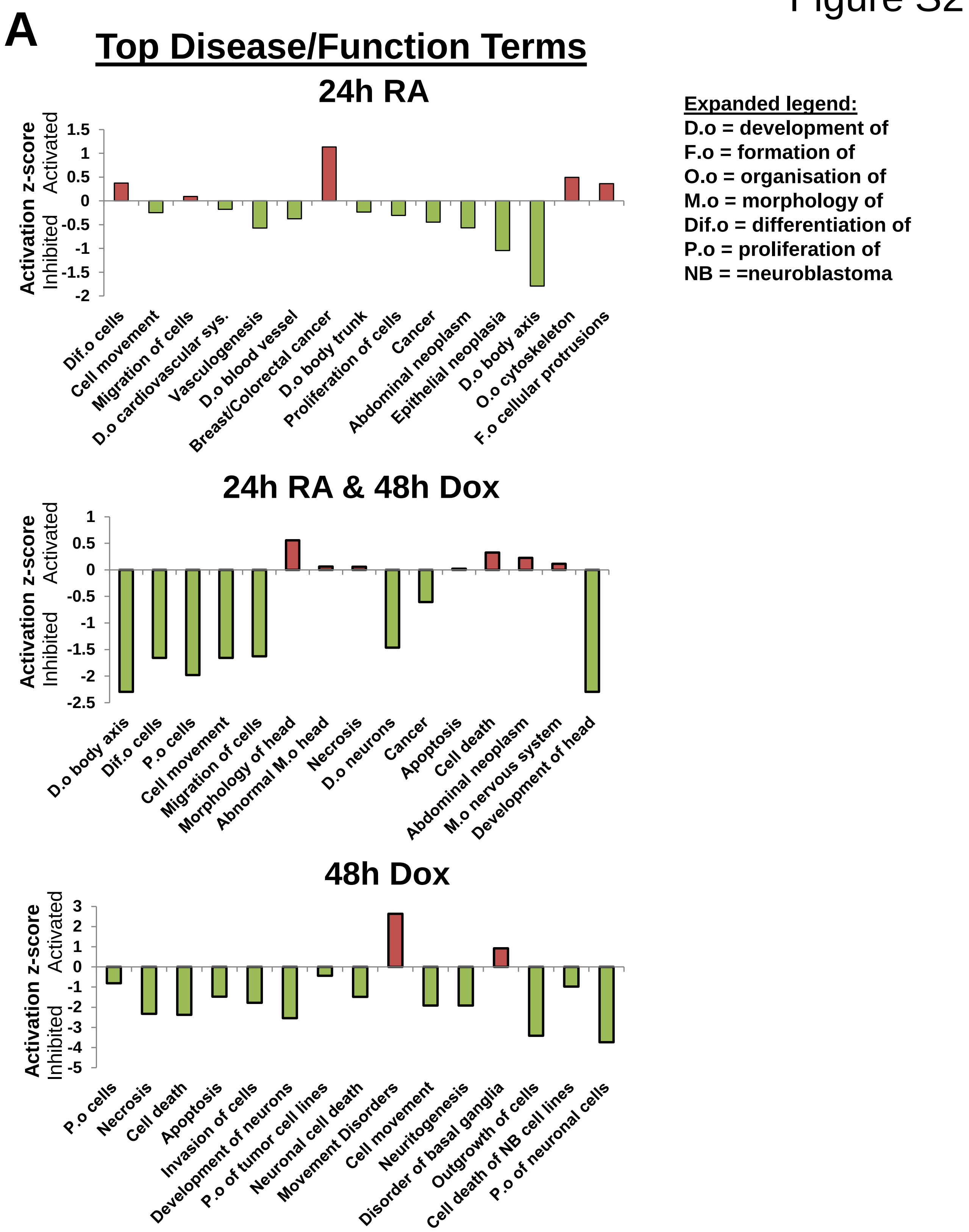

Figure S2. Additional GO term analysis for the SY5Y-MYCN RNA-seq data. (A) Activation/inhibition score of the top 15 gene ontology (GO) disease and function terms (ranked by p-value of overlap) for each of the RNA-seq treatment groups, generated by analysing the SY5Y-MYCN RNA-seq data with Ingenuity Pathway Analysis (IPA). GO term activation/inhibition levels are relative to those of the SY5Y-MYCN control cells.

Figure S3

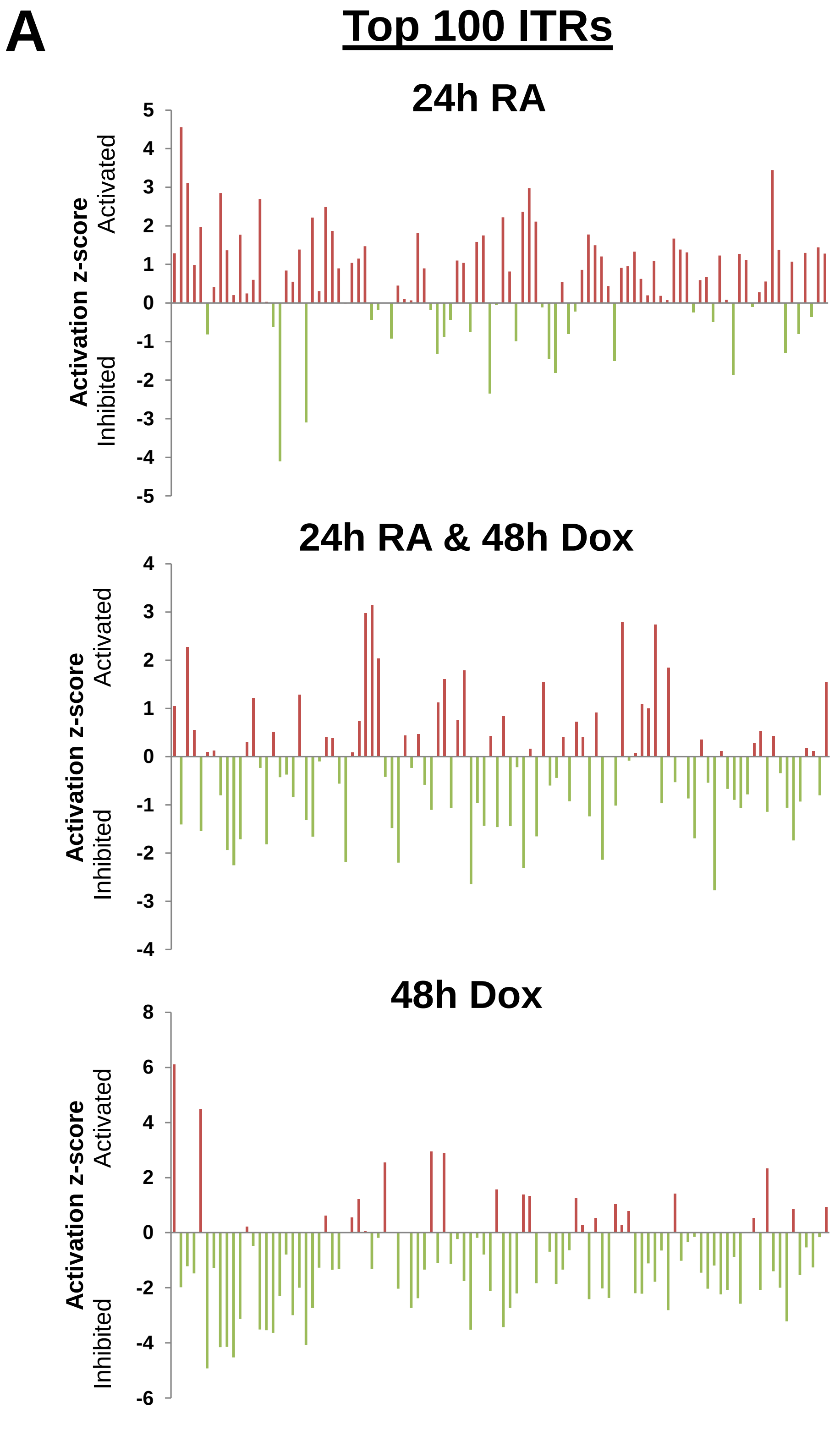

Figure S3. Additional ITR analysis for the SY5Y-MYCN RNA-seq data. (A) Overview of the activity scores (activation/inhibition) of the top 100 ITRs (ranked by p-value of overlap) from each of the three treatment groups from the SY5Y-MYCN RNA-seq dataset. Activation/inhibition levels for each ITR are relative to those of the SY5Y-MYCN control cells.

Figure S4

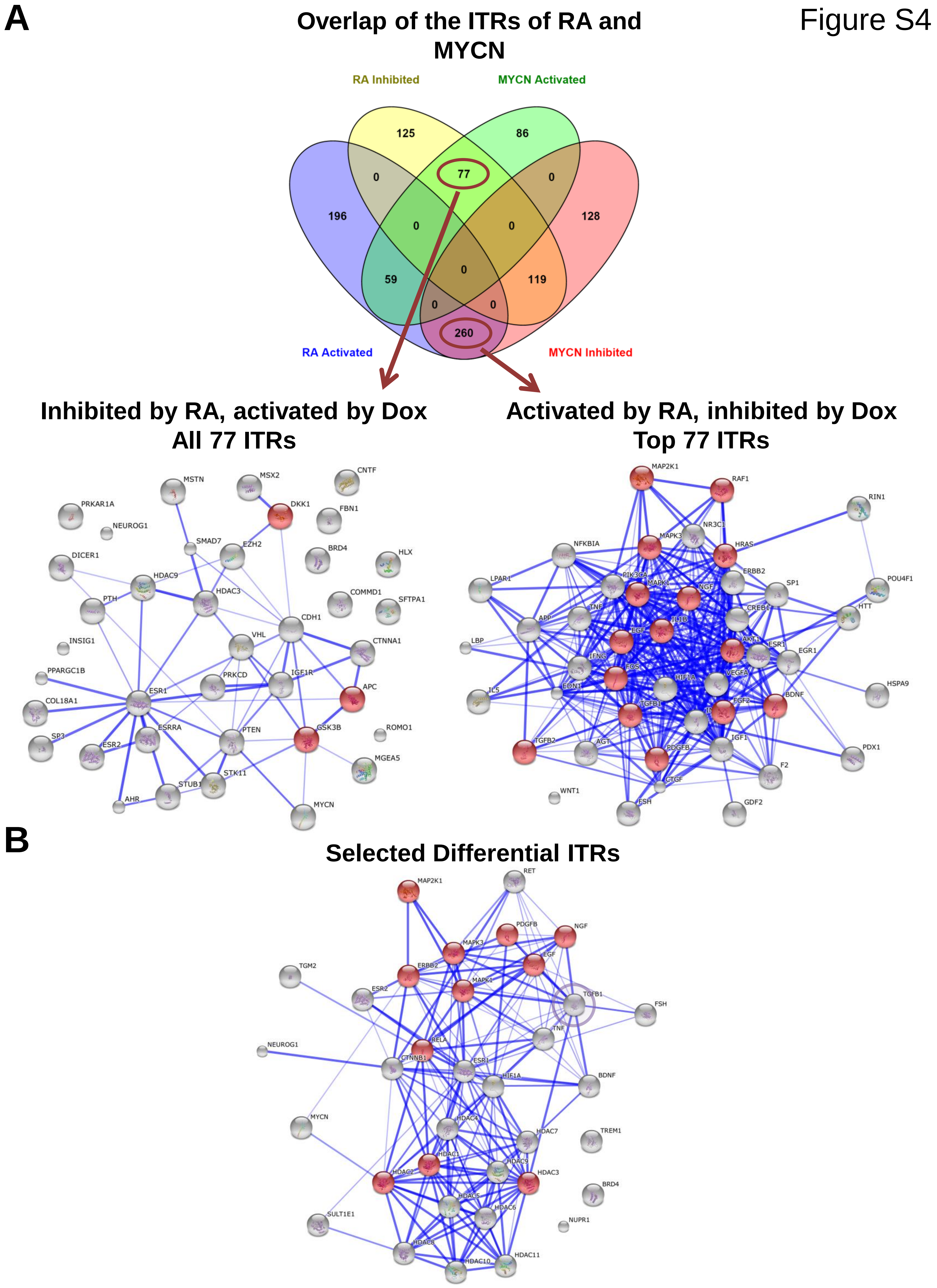

Figure S4. Additional data showing that RA and MYCN overexpression drive opposing activation dynamics of their downstream transcriptional regulators. (A) Top: Venn diagram of the overlap between all ITRs of the RA only (24h 1µM) and MYCN overexpression only (48h Dox) treatments. The ITRs for each of the two conditions have been separated into those which were activated and those which were inhibited in each treatment, to show ITRs altered in opposing directions in response to RA and MYCN overexpression. Venny (http://bioinfogp.cnb.csic.es/tools/venny/index.html) was used to generate the Venn diagram. Bottom: Protein-protein interaction map of the 77 ITRs which were inhibited by RA and activated by MYCN overexpression (left), and the top 77 (of 260) ITRs activated by RA and inhibited by MYCN (right). The protein interaction maps of previously known connections between these proteins were generated using the String database. Note: only protein ITRs are shown; miRNAs and drug compounds are excluded. Wnt (left) and MAPK (right) associated nodes, as defined by String's KEGG pathway enrichment tool, are shaded red. **(B)** Protein-protein interaction map of the selected ITRs (protein ITRs only) from Fig. 4D, showing their high degree of interconnectedness. Neurotrophin signalling pathway associated nodes, as defined by String's KEGG pathway enrichment tool, are shaded red. The TGBF1 node is circled by a purple halo.

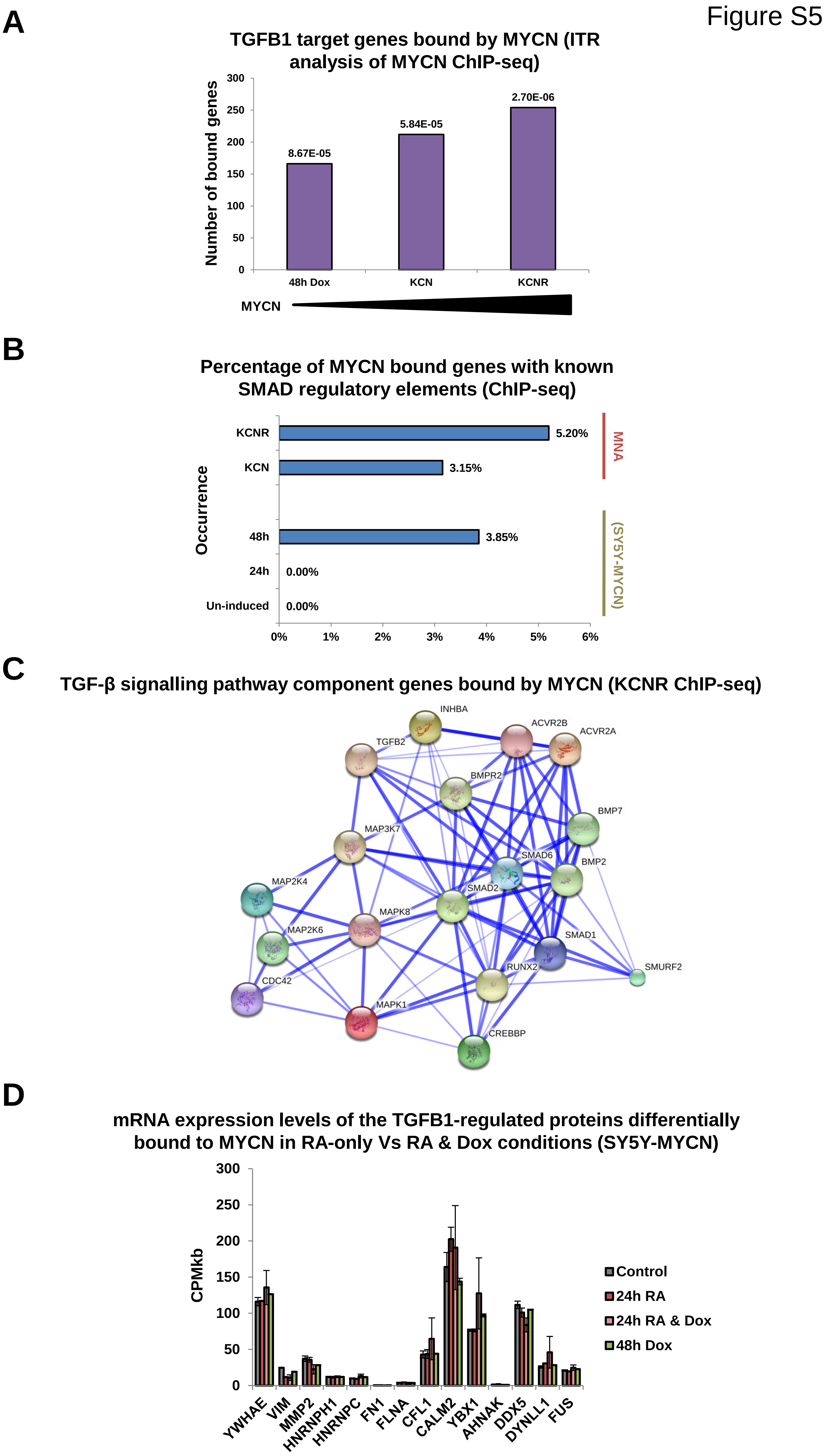

Figure S5. Additional TGF-β/SMAD MYCN ChIP-seq and interactome data. (A) Number of known TGFB1 target genes which were bound by MYCN (ChIP-seq), contributing to TGFB1 being a top predicted ITR of MYCN's genomic targets. Data from MYCN ChIP-seq from amplified MYCN (KCN, KCNR) and overexpressed MYCN (SY5Y-MYCN) cell lines. The p-value of overlap for each cell line is shown above the bars. **(B)** DiRE analysis (http://dire.dcode.org) [81] of amplified MYCN (KCN, KCNR) and overexpressed MYCN (SY5Y-MYCN) ChIP-seq data showing the percentage (occurrence) of MYCN bound genes which are also SMAD targets, i.e. known to have SMAD binding regulatory elements. **(C)** Protein-protein interaction map of the TGF-β signalling pathway component genes which were bound by amplified MYCN in the KNCR cell line, as revealed by MYCN ChIP-seq. The protein interaction maps of previously known connections between these proteins were generated with the String database. **(D)** Levels of absolute gene expression in SY5Y-MYCN cells of the TGFB1-regulated proteins differentially bound to MYCN in RA-only versus RA & Dox conditions (Fig. 5E). Expression is in read counts per million adjusted by gene length in kilobases (CPMkb), with error bars denoting the standard deviation between replicates.

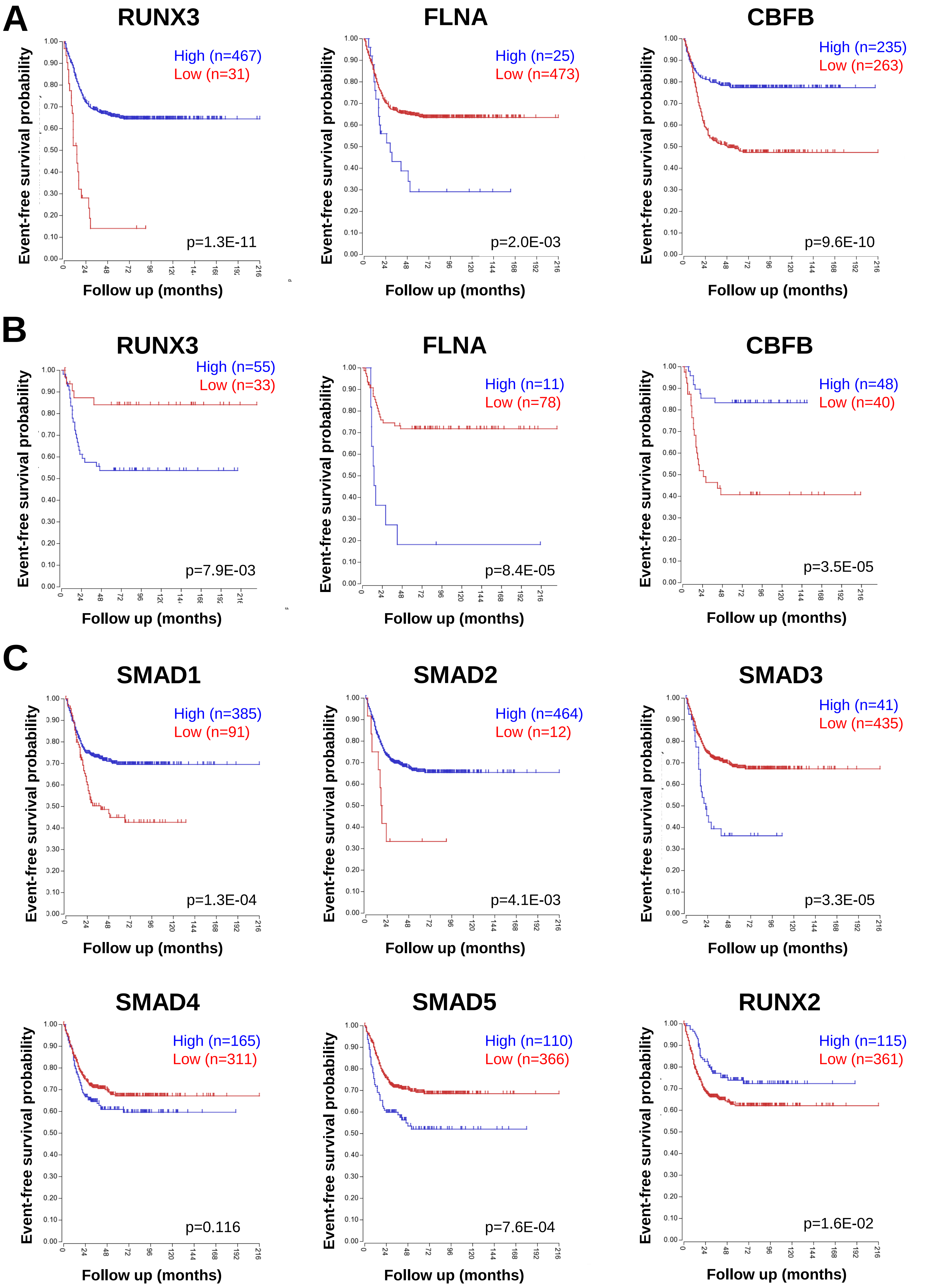

Figure S6. Additional data showing TGF-β pathway components targeted by the small molecule kartogenin are predictive of neuroblastoma patient outcome. (A, B) Kaplan-Meier survival curves showing the predictive strength of the expression levels of the RUNX3, FLNA and CBFP mRNAs in neuroblastoma tumours on patient outcome. Generated using the SEQC [68] 498 neuroblastoma tumour dataset (A) and the Versteeg [67] 88 neuroblastoma tumour dataset (B) in the R2: Genomics Analysis and Visualization Platform (http://r2.amc.nl). **(C)** Kaplan-Meier survival curves showing the predictive strength of the expression levels of the SMAD1-5 and RUNX2 mRNAs in neuroblastoma tumours on patient outcome. Generated using the Kocak [69] 649 neuroblastoma tumour dataset in the R2: Genomics Analysis and Visualization Platform (http://r2.amc.nl).

Figure S7

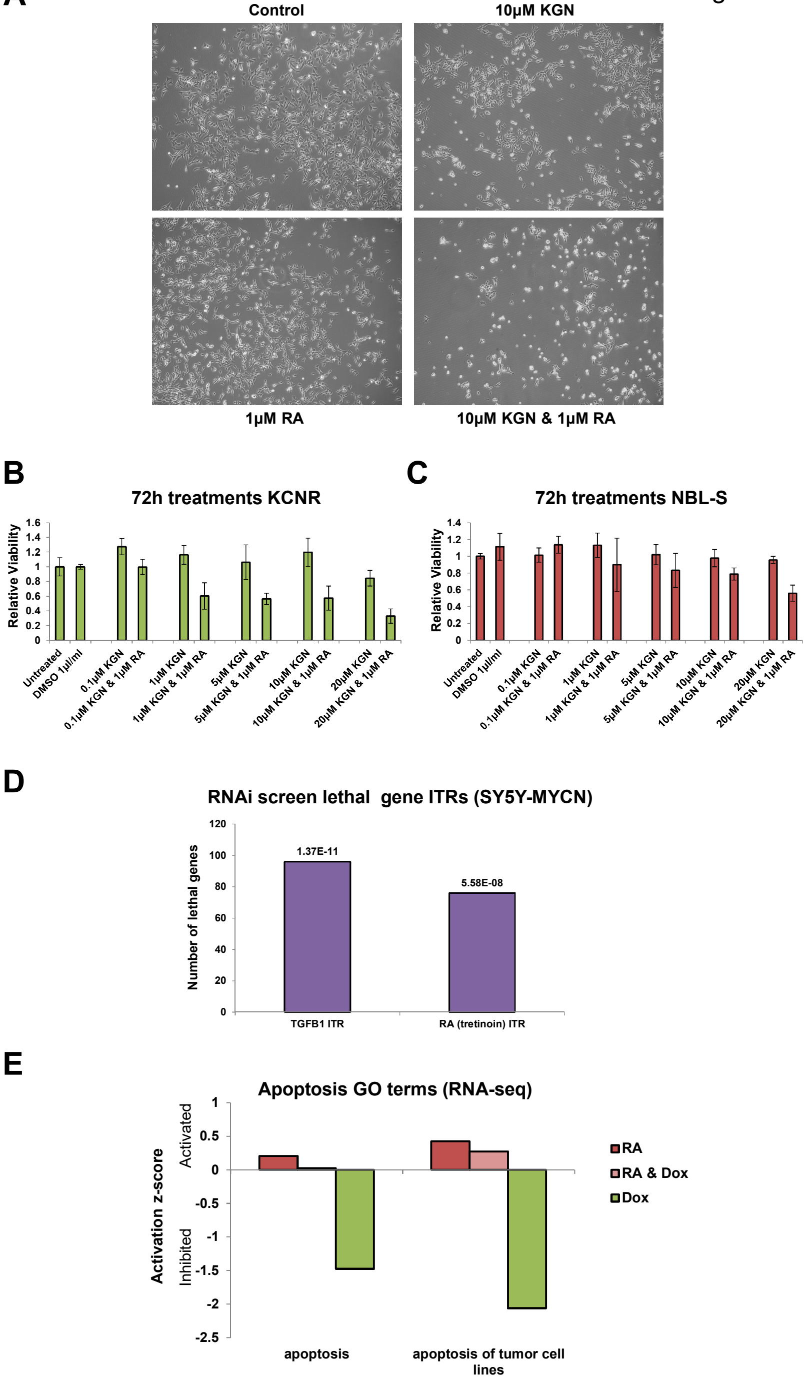

Figure S7. Additional kartogenin and retinoic acid cell viability and apoptosis data. (A) Imaging of IMR32 cells treated for 3 days with a single compound (10µM KGN or 1µM RA) or combination treatment (10µM KGN and 1µM RA), revealed a large proportion of apoptotic-like rounded cells upon combination treatment. All panels are imaged at 10x magnification. **(B, C)** Cell viability analysis of MYCN-amplified KCNR cells **(B)** or single-copy-MYCN but with elevated MYCN protein levels, NBL-S cells **(C)** treated for 72h with KGN, either individually or in combination with RA, as detected by MTS assay. Viability is set relative to that of the respective control untreated cells (KCNR or NBL-S), with error bars denoting the standard deviation between replicates. **(D)** Numbers of TGFB1 and RA associated target genes, as identified by IPA ITR analysis, which strongly (greater than 2 standard deviations from the median of the screen, 674 genes in total) reduced SY5Y-MYCN viability (in either condition) when knocked down (72h RNAi screen with druggable-genome siRNA library).The p-values of overlap between the RNAi hits corresponding to the ITR and all known target genes of that ITR are shown above each bar. **(E)** IPA disease and function GO term analysis of the SY5Y-MYCN RNA-seq samples revealed that genes differentially expressed upon 24h RA treatment (SY5Y-MYCN cells) were enriched for genes associated with the activation of apoptosis, while genes differentially expressed upon 48h of MYCN overexpression (SY5Y-MYCN cells) were enriched for genes associated with the inhibition of apoptosis. The effect of combination treatment (24h RA and 48h MYCN overexpression) was intermediate to the single agent treatments. Values are relative to those of the SY5Y-MYCN control cells.
